# Disentangling Excimer Emission from Chiral Induction in Nanoscale Helical Silica Scaffolds Bearing Achiral Chromophores

**DOI:** 10.1002/cphc.202200573

**Published:** 2022-11-04

**Authors:** Maria João Álvaro‐Martins, José Garcés‐Garcés, Antoine Scalabre, Peizhao Liu, Fernando Fernández‐Lázaro, Ángela Sastre‐Santos, Dario M. Bassani, Reiko Oda

**Affiliations:** ^1^ Área de Química Orgánica Instituto de Bioingeniería Universidad Miguel Hernández 03202 Elche Spain; ^2^ Univ. Bordeaux CNRS Bordeaux INP CBMN UMR 5248 33600 Pessac France; ^3^ Univ. Bordeaux CNRS Bordeaux INP ISM UMR 5255 33400 Talence France; ^4^ WPI-Advanced Institute for Materials Research Tohoku University Katahira, Aoba-Ku 980-8577 Sendai Japan

**Keywords:** chirality, diketopyrrolopyrrole, fluorescence, perylenemonoimidodiesters, silica nanosystems

## Abstract

The synthesis and characterization of diketopyrrolopyrroles and perylenemonoimidodiesters linked to a substituted benzoic acid in the *ortho*, *meta*, and *para* positions, are reported. Grafting of these dyes on the surface of chiral silica nanohelices is used to probe how the morphology of the platform at the mesoscopic level affects the induction of chiroptical properties onto achiral molecular chromophores. The grafted structures are weakly (diketopyrrolopyrroles) or strongly (perylenemonoimidodiesters) emissive, exhibiting both locally‐excited state emission and a broad, structureless emission assigned to excimers. The dissymmetry factors obtained using circular dichroism highlight optimized supramolecular organization between the chromophores for enhancing the chiroptical properties of the system. In the *ortho*‐ derivatives, poor organization due to steric hindrance is reflected in a low density of chromophores on walls of the silica‐nanostructures (<0.1 vs. >0.3 and up to 0.6 molecules/nm^2^ for the *ortho* and *meta* or *para* derivatives, respectively) and lower *g*_abs_ values than in the other derivatives (*g*_abs_<2×10^−5^ vs 6×10^−5^ for the *ortho* and *para* derivatives, respectively). The *para* derivatives presented a better organization and increased values of *g*_abs_. All grafted chromophores evidence varying degrees of excimer emission which was not found to directly correlate to their grafting density.

## Graphical abstract

**Grafting fluorescent dyes** onto chiral macroscopic objects can lead to chiral induction and the formation of excimers. Are these two related? Let′s find out!

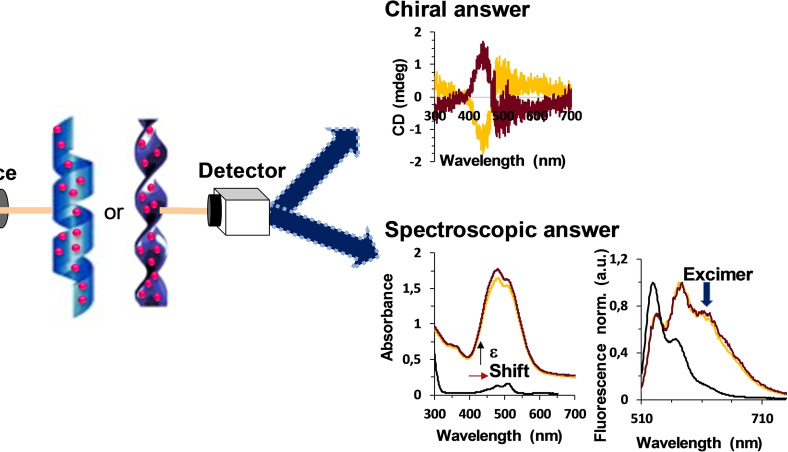

## Introduction

Chirality is a characteristic of non‐centrosymmetric 3D objects which affects how objects interact through shape complementarity. Chirality can also affect a molecule‘s reactivity and how it interacts with electromagnetic radiation. In Nature, chirality is omnipresent and used at the molecular level to direct recognition and the formation of molecular assemblies. For this reason, understanding chiral interaction between molecules has been an important area of research since the seminal work of Pasteur.^[1]^ In particular, chiral induction is the process by which a chiral object induces chiral properties to an achiral entity and represents a transfer of morphological information that is not contingent on differences in bulk physical or chemical properties between enantiomers. Chiral induction between molecules can be rationalized and designed using molecular modeling and promising results have been obtained using molecular imprinted materials. However, these approaches still require the availability of suitable chiral molecular templates. Meanwhile, the induction of chirality from chiral objects with micro‐ or macroscopic dimensions to molecules is a significantly greater challenge as this mechanism requires a transfer of information over multiple length scales that is not easily generalized or modeled.[^2^, ^3^, ^4^]

Nevertheless, chiral induction from a universal macroscopic chiral platform to molecules represents an attractive approach to impart chirality onto a wide range of bound chromophores. We have shown that robust chiral silica templates can confine surface‐bound achiral molecular guests in a chiral environment as evidenced by the appearance of chiroptical properties such as circular dichroism (CD) or circularly polarized luminescence (CPL).[^5^, ^6^] Along these lines, results supporting the induction of chiroptical signal in gold nanoparticles, in metal complexes,[^7^, ^8^, ^9^] in perovskite,^[10]^ or using biphenol‐linked polysilesquioxane through a sol‐gel transcription of self‐assembled twisted or helical ribbons from dicationic gemini surfactants have been obtained.^[11]^

Our recent efforts in elucidating how chiral silica helices impart chiroptical properties to achiral chromophores located on their periphery or interior suggest that molecular packing is an important parameter that is sometimes manifested through the formation of an emissive excimer in the case of fluorescent dyes grafted on the silica surface.^[12]^ For intramolecular multichromophore systems or systems with reduced molecular motion such as monolayers or polymers, excimer formation is governed by the population of ground‐state conformations that can form an excimer within the short (typically a few ns) lifetime of the excited state. This has been formulated as the NEER (non‐equilibration of excited rotamers) principle^[13]^ and was independently confirmed for numerous systems involving excimers.[^14^, ^15^, ^16^] In the present case, the system behaves similarly to a monolayer, and only those chromophores that are close in space in the ground state can form an excimer upon excitation. No or little ground‐state aggregation is observed since the potential energy surface between the chromophores remains repulsive. Therefore, although the excimers reflect excited‐state geometries, these are inherently related to the ground‐state geometry due to the restricted motion of the chromophores and the short excited‐state lifetime.

Herein, we explore the relationship between excimer formation among surface‐bound chromophores and chiral induction by comparing two families of chromophores (diketopyrrolopyrroles, DPPs, and perylenemonoimidodiesters, PMIDEs) with a differing propensity towards excimer formation. For each group, the position of the anchoring point (*ortho*, *meta*, or *para*) is examined. These two families of compounds were chosen due to the fact that both DPP and PMIDE possess synthetic versatility and high physical and chemical stabilities. Furthermore, they exhibit interesting optoelectronic properties, such as intense absorption in the UV‐vis and high fluorescence quantum yields,[^17^, ^18^, ^19^, ^20^, ^21^, ^22^] and have extended aromatic cores conducive towards π‐π interactions. As a consequence, both ground and excited‐state electronic interactions between chromophores are expected to be favored upon grafting to chiral silica nanostructures.[^23^, ^24^, ^25^]

## Results and Discussion

**Figure 1:**
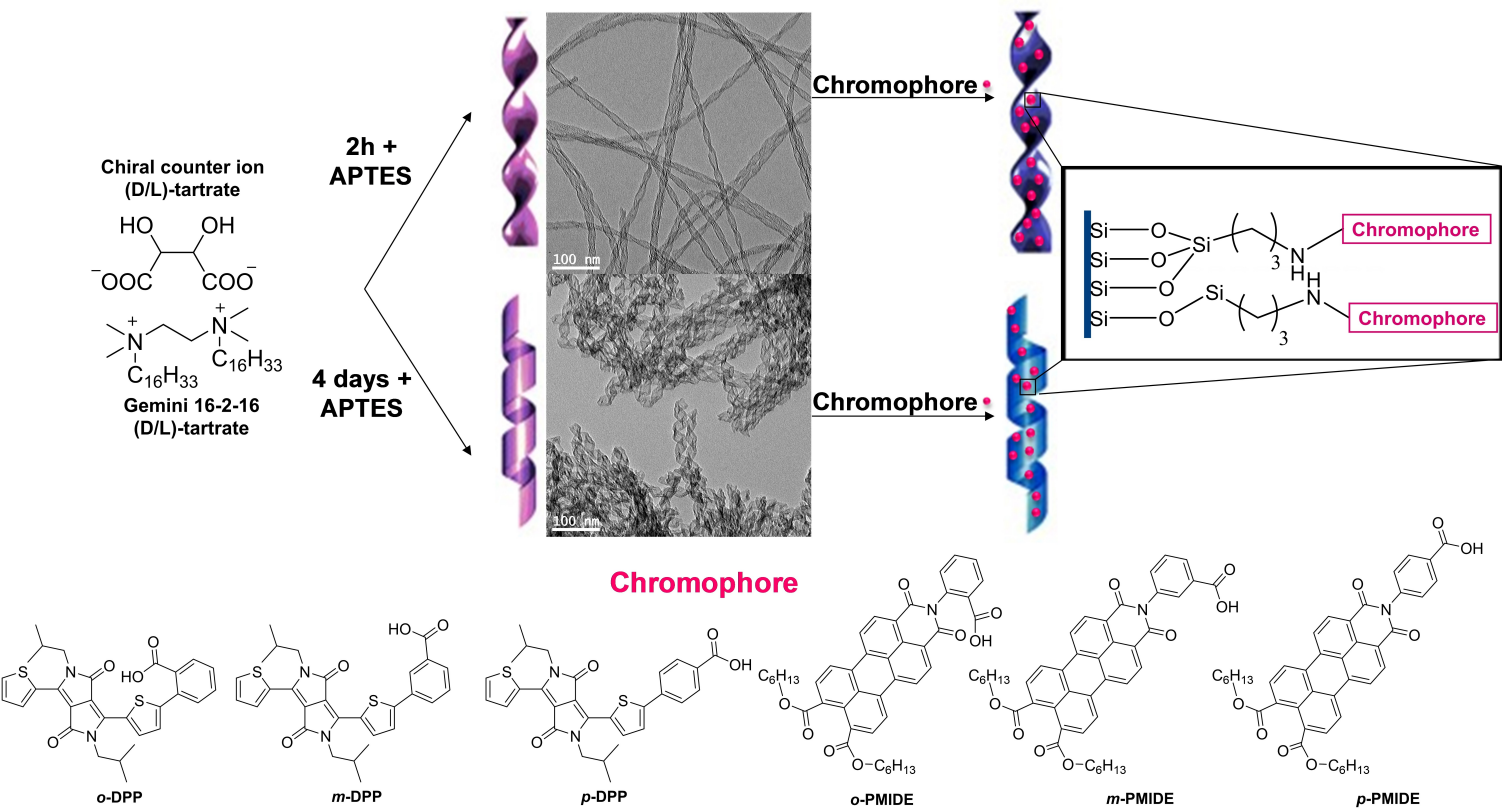
Preparation process of silica nanostructures and respective TEM images, along with the structure of the achiral chromophores used in grafting.

The DPP and PMIDE chromophores selected for the study are shown in Figure 1. They were grafted onto chiral twisted or helical silica ribbons through amide coupling of the benzoic acid group to the silica surface previously functionalized by (3‐aminopropyl) triethoxysilane.[^9^, ^26^] The synthesis of the organic chromophores is described in the Supporting Information. The silica nanostructures were synthesized according to the previously reported protocols and the morphology of the nanostructures was verified by transmission electron microscopy (TEM) (Figure 1).^[27]^

While the global shape of the absorption spectra of the benzoic‐acid substituted DPP and PMIDE chromophores in methanol and in chloroform solutions, respectively, are generally similar to those of the parent compounds, the former present a 15–25 nm bathochromic shift due to extension of the conjugation across the benzoic acid moiety. Strong hypochromism can be observed for ***p*** **‐PMIDE** (15351 *vs* 56424 M^−1^cm^−1^ for ***p*** **‐PMIDE** and **PMIDE‐Ref**, respectively, see supplementary information) which is assigned to the formation of *J*‐aggregates in solution. All the compounds are emissive, and the salient photophysical properties are summarized in Table 1 (see also Figure S1 in the Supporting Information).

**Table 1:** Absorption and emission properties of DPPs^[a]^ and PMIDEs.^[b]^

Compound	λ_abs_ [nm]	ϵ [M^−1^cm^−1^]	λ_em_ [nm]	Φ_F_	τ [ns]
***o*** **‐DPP**	555	14396	590	0.071	2.1/3.3
***m*** **‐DPP**	562	27540	594	0.046	2.1/4.1
***p*** **‐DPP**	564	31150	602	0.053	1.9/3.6
***o*** **‐PMIDE**	505	52584	526	0.23	4.7
***m*** **‐PMIDE**	508	56436	528	0.16	4.6
***p*** **‐PMIDE**	505	15351	529	0.84	4.3

[a] Methanol solution. [b] Chloroform solution.

**Figure 2:**
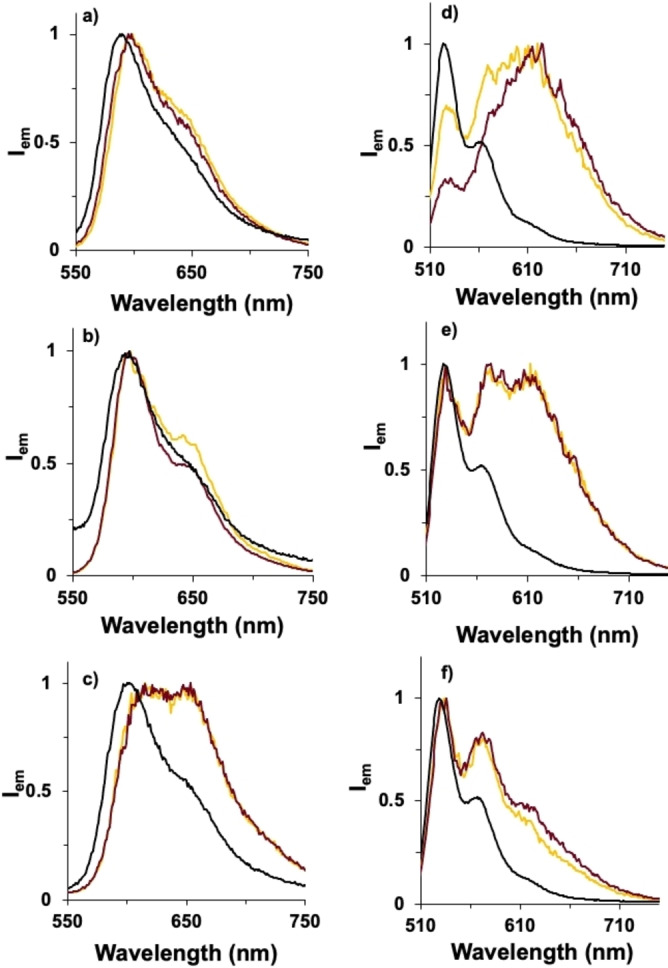
Normalized fluorescence emission of a) ***o*** **‐DPP**‐, b) ***m*** **‐DPP**‐, c) ***p*** **‐DPP**‐grafted helical ribbons (λ_ex_=530 nm, methanol) and d) ***o*** **‐PMIDE**‐, e) ***m*** **‐PMIDE**, f) ***p*** **‐PMIDE**‐grafted helical ribbons (λ_ex_=510 nm, chloroform). Black lines correspond to free dyes, yellow and red lines to the dyes grafted on the surface of right‐ and left‐handed helical ribbons, respectively. See Figures S4–S5 for the emission spectra from twisted ribbons.

The dyes were grafted as benzamides onto the amine‐decorated silica structures via their mixed anhydride. Purification was achieved by successive washing and centrifugation to isolate the silica architectures from the reagents and unreacted dye. In all cases, the grafting onto silica twisted or helical ribbons was evidenced by absorption and emission spectra compared with the grafted dyes (Figures 2 and 4 and S2–S5). No changes in the silica architecture's morphology could be seen by TEM imaging (Figures S6–S8), confirming that these were not adversely affected by the grafting conditions.

**Figure 3:**
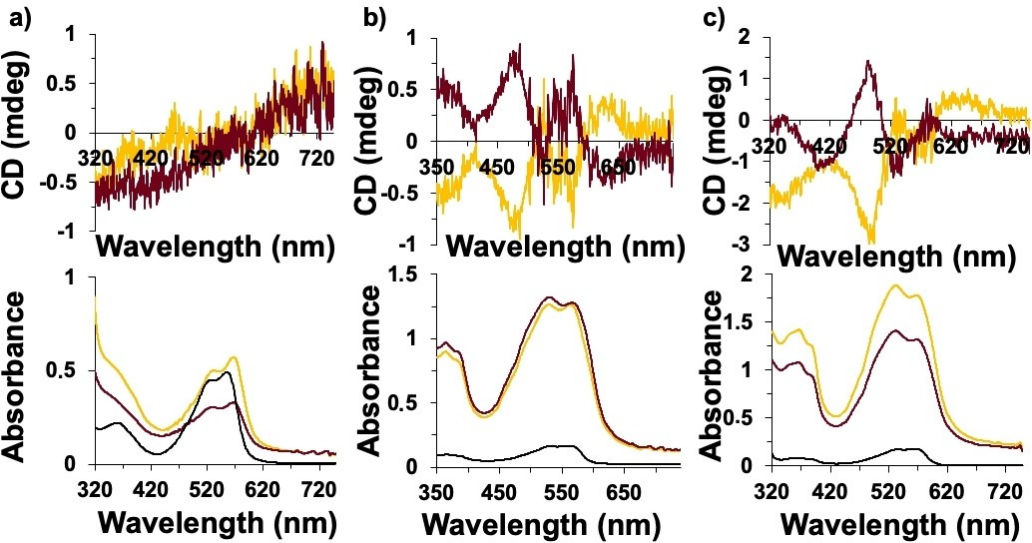
CD (top) and UV‐vis spectra (bottom) in methanol of a) ***o*** **‐DPP**, b) ***m*** **‐DPP**, and c) ***p*** **‐DPP** before and after grafting on the surface of helical ribbons. Black lines correspond to dyes‐free, yellow and red lines correspond to the dyes grafted on the surface of right‐ and left‐handed helical ribbons, respectively.

**Figure 4:**
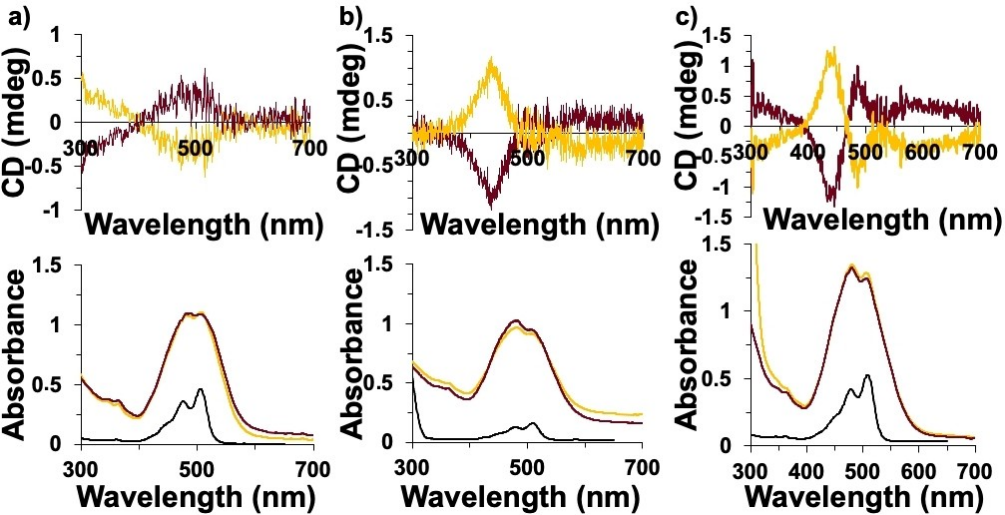
CD (top) and UV‐vis spectra (bottom) in chloroform of a) ***o*** **‐PMIDE**, b) ***m*** **‐PMIDE**, and c) ***p*** **‐PMIDE** before and after grafting on the surface of helical ribbons. Black lines correspond to dyes‐free, yellow and red lines correspond to the dyes grafted on the surface of right‐ and left‐handed helical ribbons, respectively.

In methanol, the absorption spectra of the DPP‐ helical or – twisted ribbon nanostructures are broader and slightly red‐shifted compared to the corresponding free dye in solution. This evidences the occurrence of some ground‐state electronic coupling between the organic units when grafted onto the nanostructure surface. Concomitantly, the emission spectra of the DPP‐helical or ‐twisted ribbons showed the presence of an additional broad, structureless feature at 650 nm that is assigned to excimer emission.^[28]^ Relative to the locally‐excited emission, the contribution of the excimer follows the order *para*>*meta*>*ortho*. As a result, the emission decay is multi‐exponential, with a short component of 1.5–2.1 ns and a longer contribution of 3.8–4.8 ns (see S.I. for details). A similar situation is observed for the PMIDE‐grafted twisted or helical ribbons, albeit with stronger emission and longer excited‐state decays (3.9 ns and 16–20 ns for the short and long contributions, respectively). However, in contrast to the DPP series, the contribution of the broad, longer wavelength assigned to the excimer is more important for ***o*** **‐PMIDE** and follows the order *ortho*>*meta*>*para* (Figure 2).

Chiral induction from the inorganic template to the achiral chromophores was probed using induced circular dichroism (ICD). It is assumed that an achiral molecule grafted in the immediate vicinity of a chiral surface will probe the chirality of the environment by combining the direct effects of the chiral curvature of the surface and the chiral object (solvent, molecular organization). Particularly for this latter reason, as noted previously, it is important to take into account the grafting density of the dyes when comparing ICD and excimer emission.^[12]^ The grafting density of the dyes was calculated and is summarized in Table 2 for both right‐ and left‐handed silica helical or twisted ribbons.^[29]^

**Table 2:** Grafting density and chiroptical properties of DPPs and PMIDEs.

Compound	Twisted ribbons		Helical ribbons	
	η [L/D]^[a]^	10^5^ **⋅** *g*_abs_ [nm]	η [L/D]^[a]^	10^5^ **⋅** *g*_abs_ [nm]
***o*** **‐DPP**	0.09/0.12	0	0.07/0.04	0
***m*** **‐DPP**	0.27/0.27	2.8 (473)	0.30/0.30	3.2 (474)
***p*** **‐DPP**	0.36/0.58	5.2 (488)	0.62/0.46	6.3 (484)
***o*** **‐PMIDE**	0.07/0.07	0	0.08/0.08	1.2 (497)
***m*** **‐PMIDE**	0.18/0.18	3.8 (444)	0.16/0.16	4.5 (445)
***p*** **‐PMIDE**	0.55/0.55	4.4 (437)	0.35/0.35	5.3 (435)

[a] Grafting density in molecules/nm^2^ for left (D) and right (L) handed twisted and helical ribbons.

The results show that the *ortho*‐substituted benzamides have much lower surface coverage than the corresponding *meta‐* or *para‐*derivatives. This can be understood on the basis of steric interactions that constrain the geometry of the benzamide linker to be perpendicular to the plane of the aromatic chromophore. As a consequence, the number of available surface binding sites would be restricted to those able to accommodate these added constraints. Higher and more uniform grafting densities are obtained with the *meta*‐ and *para*‐substituted benzamide groups, with the latter providing the highest grafting densities for both the DPP and PMIDE series. Interestingly, while this results in a decrease of the proportion of excimer emission in the DPP series, the strongest excimer emission is observed for ***o*** **‐PMIDE** instead at similar grafting densities, which then decreased with the increasing grafting density. This may indicate that the latter undergoes localized aggregation on the surface due to the propensity of the PMIDE chromophore to form excimers given sufficient conformational mobility as previously observed for a pyrene‐acetic acid derivative or the aggregation‐induced quenching observed by PMIDE.^[12]^

The induction of chiroptical properties was assessed for all the assemblies by measuring the induced circular dichroism and calculating the absorption dissymmetry factor (*g*_abs_) according to Equation (1), where ▵*A*_CD_ is the difference in absorption of left and right circularly polarized light and *A* is the absorbance of the sample at the same wavelength.

(1) $$g_{abs}=\frac{{\Delta A}_{CD}}{A}$$

For both series, the *g*_abs_ are directly related to the grafting density, and follow the order *g*_abs_ *para>g*_abs_ *meta>g*_abs_ *ortho* for both dyes and for both twisted and helical ribbons (Table 2 and Figures 3–4). The grafting density can be estimated from the amount of chromophore present in the sample (determined by electronic absorption spectroscopy) compared to the surface of the inorganic architectures (estimated from their dimensions as determined from TEM).^[29]^ For the ***o*** **‐PMIDE** derivative, strong excimer emission suggests that the chromophore is more densely grafted in localized regions of the helices, possibly explaining the small chiral induction observed for this derivative in the case of the helical architectures.

Both silica twisted and helical ribbons present morphological chirality as expressed by their surface curvature. However, there are subtle differences between the two morphologies:^[29]^ In the case of helical ribbons, the curvature is defined by the pitch and diameter of the helix and is invariant with respect to the positioning of a chromophore on the surface of the helical ribbons. For twisted ribbons, the morphology is instead characterized by a twisting angle that increases as one moves from the exterior towards the center of the ribbon. This, combined with a greater shrinkage during the formation of the helical *vs*. twisted ribbon morphologies, can explain the larger chiral induction observed for helical architectures.^[30]^ A comparison of the *g*_abs_ data in Table 2 evidences that for both the DPP and PMIDE series of chromophores, the chiral induction observed upon grafting to helical ribbons is indeed larger than that observed upon grafting to twisted ribbons. This observation also holds within each series when comparing the effect of the position of the anchoring point. Interestingly, even though the ***o*** **‐DPP** derivative grafts onto twisted ribbons with a larger density than the other *ortho*‐substituted derivatives, no chiral induction is observed.

The results highlight the complexity of the relationship between surface binding, excimer emission, and induction of chirality. For both series, the point of attachment determines the grafting density. However, the DPP and PMIDE series offer contradictory trends, with the former showing an increase in excimer emission with increasing surface density whereas the latter shows the highest excimer emission for the lowest surface density.

## Conclusions

We find that all six achiral benzoic acid‐based on DPP and PMIDE cores, (***o*** **‐DPP**, ***m*** **‐DPP**, ***p*** **‐DPP**, ***o*** **‐PMIDE**, ***m*** **‐PMIDE**, and ***p*** **‐PMIDE**) exhibit excimer emission upon grafting onto chiral silica helical and twisted ribbons through amide coupling. Their grafting density is directly determined by the point of attachment, with *η*(*para*)>*η*(*meta*)>*η* (*ortho*) for both the DPP and PMIDE series on either ribbons or helices. Although in general excimer emission is expected to be dependent on grafting density (as in the DPP series), we find that the contrary is true for the PMIDE series. Here, the ***o*** **‐PMIDE** derivative with the lowest grafting density shows the highest excimer contribution. This can be rationalized on the basis of the propensity for the perylene chromophore to form excimers given sufficient conformational mobility. The higher grafting densities of the ***m***‐ and ***p*** **‐PMIDE** derivatives may result in less conformational freedom leading to lower excimer formation yields from the localized excited state.

The induction of chiroptical properties was studied by induced circular dichroism and quantified by the dissymmetry factor. For both series of chromophores, the results indicate that the density of chromophores on the surface of the silica nanostructures is directly correlated with the value of g_abs_. The lowest values of grafting density were obtained for the derivatives substituted in the *ortho‐* position, resulting in low g_abs_ values. From previous results, we conclude that, insomuch as excimer emission can be related to surface density, it provides an indirect correlation to the induced chiroptical properties. However, this relationship is tenuous since systems in which conformational motion is needed to favor excimer formation (e. g. bulky chromophores) will show the reverse relationship. This is the case for the PMIDE series, in which chiroptical induction and excimer emission intensity are negatively correlated.

## Experimental section

Full experimental details and methods for the synthesis and characterization of achiral dyes are described in the supporting information.

**Grafting of the dyes on the surface**. The grafting of the dyes on the surface takes place in two steps. The first is the activation of the carboxylic acid of the dyes to react with the primary amino groups present on the surface of silica nanostructures.[^9^, ^26^] The acid derivatives were dissolved into 4 mL of an aprotic solvent (dried acetone for ***o*** **‐DPP** and **PMIDE** derivatives or DMF for ***p*** **‐DPP** and ***m*** **‐DPP**), then 2 equivalents of ethyl chloroformate and 2.5 equivalents of triethylamine were added under nitrogen atmosphere. The reaction was left at rt for 3 h. Meanwhile, the APTES‐modified silica twisted or helical ribbons were washed five times with the solvent used in the acid activation. Then, without further purification, the solution of activated dye is introduced in a vial containing the nanostructures dispersed in the respective solvent (1 μmol of chromophores per mg of nanostructure). The reaction was allowed to proceed overnight on a shaker. Then, the samples were washed several times with acetone or DMF using a centrifuge (10 min, 9800 G, rt) until all free dye was removed, and no colour is observed in the supernatant. This process was repeated to fully react to all available amine sites. Finally, 3 washes were done in methanol for DPP and in CHCl_3_ for PMIDE to remove DMF and acetone from the systems.

**Supramolecular characterization**: TEM images were collected on a CM120 (Philips) microscope or an LVEM5 instrument from Delong Instruments (Brno, Czech Republic). The instrument is working with a Schottky field emission gun operating at a nominal acceleration voltage of 5 kV, and a crystal of yttrium aluminium garnetis (YAG) is used to convert the electronic image into a visible image, which is observed using a camera (Zyla 5.5 Scientific CMOS) through a microscope objective (Objectives Olympus M4× or 40×). Circular dichroism spectra were measured on a Jasco J‐815 (190–800 nm) instrument equipped with a xenon‐mercury lamp and running under a nitrogen atmosphere. The sample temperature was maintained at 20 °C using a Peltier device (PFD‐425S/15). All samples were stirred at 1000 rpm during measurements, and every spectrum was taken as the average of 4–10 scans. As well as the CD spectra, the fluorescence spectra were also performed under agitation. Fluorescence spectra were measured on a Fluormax‐4 spectrophotometer. Lifetime measurements were performed on a PicoQuant PDL 800‐D. The excitation originated from a diode laser at 450 nm operated in pulsed mode.

## Supporting Information Summary

Full details of the synthesis of the DPP and PMIDE derivatives used in this study, their characterization (^1^H and ^13^C NMR, HRMS, FTIR, excited‐state lifetime, absorption and emission spectra), and grafting on the silica helical or twisted ribbons (CD, TEM, lifetimes) are available in the supplementary information. Additional references cited within the Supporting Information.[^31^, ^32^, ^33^]

## Conflict of interest

The authors declare no conflict of interest.

1

## Supporting information

As a service to our authors and readers, this journal provides supporting information supplied by the authors. Such materials are peer reviewed and may be re‐organized for online delivery, but are not copy‐edited or typeset. Technical support issues arising from supporting information (other than missing files) should be addressed to the authors.

Supporting Information

## Data Availability

The data that support the findings of this study are available from the corresponding author upon reasonable request.
