# Supplementary material for: Disentangling Excimer Emission from Chiral Induction in Nanoscale Helical Silica Scaffolds Bearing Achiral Chromophores
Source: Chemphyschem. 2022 Nov 4;24(3):e202200573. doi: 10.1002/cphc.202200573 (PMC10099559; doi:10.1002/cphc.202200573)
Supplement: Supplementary file 1 — Supporting Information [file CPHC-24-0-s001.pdf]

ChemPhysChem

Supporting Information

Disentangling Excimer Emission from Chiral Induction in Nanoscale Helical Silica Scaffolds Bearing Achiral Chromophores

Maria João Álvaro-Martins, José Garcés-Garcés, Antoine Scalabre, Peizhao Liu, Fernando Fernández-Lázaro,* Ángela Sastre-Santos,* Dario M. Bassani,* and Reiko Oda*

Index

1.1. Experimental section 2

1.1.1. Material and methods 2

1.2. Synthesis 2

1.2.1. Synthesis of DPP derivatives 2

1.2.2. Synthesis of PMIDE derivatives 4

1.3. Optical Properties 6

1.4. Characterization of nanostructures 6

1.4.1. CD and optical characterization 6

1.4.2. TEM Characterization 8

1.4.3. Lifetime Characterization 10

1.5. Structural Characterization 18

1.5.1. Characterization of the DPP derivatives 18

1.5.2. Characterization of PMIDE derivatives 27

References .... Error! Bookmark not defined.

1.1. Experimental section

1.1.1. Material and methods

All chemicals and solvents were purchased from Sigma Aldrich (Merck) and TCI and were used without further purification unless otherwise stated. Column chromatography was performed with SiO_2 (40-63 μm). NMR data (^{1}H and ^{13}C) were recorded at 25 °C with a Bruker AC300 and Bruker AC400 spectrometers. Matrix assisted laser desorption/ionization time-of-flight (MALDI-TOF) mass spectra were obtained on a Bruker Microflex LRF20 instrument using PEG as a matrix. UV-vis spectra in methanol solution were measured with a Helios Gamma spectrophotometer and a PekinElmer UV/VIS/NIR Lamba 750 spectrometer and the extinction coefficients were calculated using the Lambert-Beer Law. Fluorescence spectra were measured on a Fluormax-4 spectrophotometer. Quantum yields were determined from corrected emission spectra using Rhodamine 6G (FQY = 0.95 in MeOH EtOH) for DPP derivatives and with the integrating sphere for PMIDE derivatives. FTIR spectra were measured with a Nicolet Impact 400D spectrophotometer.

1.2. Synthesis

1.2.1. Synthesis of DPP derivatives

As shown in **Scheme S1a**, the DPP acid derivatives (***o*-DPP**, ***m*-DPP**, and ***p*-DPP**) were synthesized by Suzuki-Miyaura coupling reactions between the **DPP-Br** and the respective benzoic acid derivatives **1**, **2** and **3**, with yields of 3%, 46%, and 55%, respectively. The final compounds did not present a good solubility in common organic solvents, being partially soluble in CH_2Cl_2, $CHCl_3$, DMF, and methanol, however, ***o*-DPP** showed better solubility in these solvents than the other derivatives. The three chemical structures were confirmed by ^{1}H NMR (**Figures S31, S34,** and **S37**), FTIR spectroscopy (**Figures S32, S35,** and **S38**), and HR-MALDI-TOF mass spectrometry (**Figures S33, S36,** and **S39**). Compounds **DPP-Ref** and **DPP-Br** were synthesized according to the procedures described in the literature[31] and characterized (^{1}H NMR, ^{13}C NMR, FTIR, HR-MALDI-TOF mass spectrometry, **Figures S23-S30**, UV-vis, and fluorescence spectra).

Synthesis of DPP-Ref. Dry DMF (20 mL) was added in a two-neck flask under nitrogen that contained **DPP-H₂** (2.0 g, 6.6 mmol) and Cs_2CO_3 (6.5 g, 20 mmol) and the mixture was set at 120 ˚C. Once this temperature was reached, a solution of 1-bromo-2-methylpropane (2.7 g, 20 mmol) in dry DMF (10 mL) was added dropwise and the reaction was left stirring for 24 hours. A HCl 2M solution was added after the reaction was cooled to rt, and the mixture was extracted with ethyl acetate. Then the organic layer was dried over anhydrous $MgSO_4$ and the solvent was removed off under reduced pressure. The crude product was purified through a silica gel column with toluene as eluent to give a red solid with a 49% yield. **^{1}H-NMR:** (300 MHz, $CDCl_3$) δ= 8.98 (dd, *J* = 3.9 Hz, *J* = 1.2 Hz, 2H), 7.63 (dd, *J* = 5.1 Hz, *J* = 1.2 Hz, 2H), 7.29-7.26 (m, 2H), 3.96 (d, *J* = 7.5 Hz, 4H), 2.13 (m, 2H), 0.97 (d, *J* = 6.6 Hz, 12H). **^{13}C-NMR:** ($CDCl_3$): δ (ppm) 160.8, 139.4, 134.7, 129.7, 129.0, 127.6, 106.9, 47.8, 28.4, 19.1. **UV-vis** (MeOH): λ_{max}, nm (log ε) 340 (3.79), 506 (4.09), 539 (4.12). **FTIR** (KBr) v_{max}, cm^{-1}: 3097, 3084, 2957, 2984, 2864, 1671, 1569, 1506, 1454, 1420-1402, 1305, 1095, 1068, 815-734. **HR-MS (MALDI-TOF):** 413.1368 m/z cal. for $C_{29}H_{28}N_2O_4S_2$ (found for $[M+H]^+$ 413.1352).

Synthesis of DPP-Br. 1 mL of acetic acid was added to a solution of **DPP-Ref** (300 mg, 0.730 mmol) in chloroform (20 mL) at 5 ˚C, and later a *N*-bromosuccinimide (NBS, 129 mg, 0.730 mmol) solution in acetic acid (2 ml) was add dropwise. Allowed to reach rt, the reaction mixture was stirred for to 2h. Then, the product was washed with NaOH and H_2O, extracted with $CHCl_3$ and dried with Na_2SO_4. The final product was purified in a chromatographic column using toluene as eluent and obtaining 257 mg (72%) as a purple powder. **^{1}H-NMR:** (400 MHz, $CDCl_3$) δ= 8.89 (dd, *J* = 4.0 Hz, *J* = 1.2 Hz, 1H), 8.72 (d, *J* = 5.6 Hz, 1H), 7.64 (dd, *J* = 5.2 Hz, *J* = 1.6 Hz, 1H), 7.29-

7.26 (m, 1H), 7.23 (d, *J* = 5.6 Hz, 1H), 3.94 (d, *J* = 10 Hz, 2H), 3.87 (d, *J* =10 Hz, 2H), 2.19-2.03 (m, 2H), 0.99-0.95 (m, 12H). **^{13}C-NMR:** ($CDCl_3$): δ (ppm) 161.7, 161.5, 140.8, 139.9, 135.9, 133.6, 1321.6, 131.4, 131.0, 120.0, 128.7, 118.9, 108.1, 107.8, 48.9, 29.5, 29.4, 20.1. **UV-vis** (MeOH): λ_{max}, nm (log ε) 346 (4.10), 513 (4.37), 547 (4.39). **FTIR** (KBr) v_{max}, cm^{-1}: 3076, 2956, 2928, 2867, 1654, 1558, 1504, 1449, 1067, 817-731.**HR-MS (MALDI-TOF):** 491.0487 m/z cal. for $C_{29}H_{28}N_2O_4S_2$ (found for $[M+H]^+$ 491.0457).

Synthesis of DPP acid derivatives. To a 25 mL round-bottom flask, the **DPP-Br** (100 mg, 0.20 mmol), the corresponding benzoic acid (75.9 mg, 0.31 mmol) and tetrabutylammonium bromide (TBAB, 3.2 mg, 0.01 mmol) were added under nitrogen. Then, 5 mL of degassed dioxane were added to the flask and the solution was degassed again. After that, a degassed K_2CO_3 2 M solution (1 mL), and $Pd_2(dba)_3$ (18 mg, 0.02 mmol) solution in dioxane (6 mL) were added to the reaction flask. The reaction was stirred overnight at 90 ˚C. After that, the crude was washed with 2M HCl and water and extracted with chloroform. The compounds were purified by silica gel column chromatography using different ratios of $CHCl_3$/methanol as eluent, yielding dark pink solids.

- ***o*-DPP.** Yield: 3%. **^{1}H NMR** (400 MHz, CD_3OD): δ (ppm) 8.90-8.89 (m, 1H), 8.84 (d, *J* = 4.0 Hz, 1H), 7.98 (d, *J* = 4.0 Hz, 2H), 7.61-7.57 (m, 2H), 7.50-7.45 (m, 3H), 7.35-7.32 (q, *J* = 4.0 Hz,1H), 3.99-3.96 (m, 4H), 2.17-2.04 (m, 2H), 0.98-0.94 (m, 12H). **FTIR** (KBr): v_{max}, cm^{-1}: 3511, 3107, 2962, 2870, 1700, 1644, 1555, 1433-1403, 816-735. **UV-vis** (MeOH): λ_{max}, nm (log *ε*): 295 (3.99), 362 (3.81), 527 (4.12), 555 (4.16).**HR-MS (MALDI-TOF)**: 533.1565 m/z cal. for $C_{29}H_{28}N_2O_4S_2$ (found for $[M+H]^+$ 533.1563).
- ***m*-DPP.** Yield: 55%. **^{1}H NMR** (400 MHz, CD_3OD + 2 drops TFA-d): δ (ppm) 8.88-8.87 (m, 2H), 8.39 (s, 1H), 8.03 (t, *J* =12.0 Hz, 2H), 7.91 (d, *J* = 4.0 Hz, 2H), 7.72 (d, *J* = 4.0 Hz, 1H), 7.61-7.54 (m, 1H), 7.34 (t, *J* = 8.0 Hz, 1H), 4.02-3.94 (m, 4H), 2.06-1.99 (m, 2H), 1.05-0.95 (m, 12H). **FTIR (KBr)** v_{max}, cm^{-1} : 3448, 3078, 2961, 2930, 2869, 1725, 1689, 1664, 1555, 1420-1389, 815-734. **UV-vis** (MeOH), λ_{max}, nm (log *ε*): 305 (4.22), 365 (4.12), 531 (4.48), 561 (4.49). **HR-MS (MALDI-TOF)**: 533.1563 m/z cal. for $C_{29}H_{28}N_2O_4S_2$ (found for $[M+H]^+$ 533.1563).
- ***p*-DPP.** Yield: 46%. **^{1}H NMR** (400 MHz, CD_3OD): δ (ppm) 8.93 (d, *J* = 4.0 Hz, 1H), 8.87 (d, *J* = 4.0 Hz, 1H), 8.04 (d, *J* = 12.0 Hz, 2H), 7.91(d, *J* = 4.0 Hz, 2H) 7.80 (d, *J* = 12.0 Hz, 2H), 7.72 (d, *J* = 8.0 Hz, 1H), 7.35 (t, *J* = 8.0 Hz, 1H), 4.01(dd, *J* = 12.0 Hz, *J* = 3.0 Hz, 4H), 2.22-2.11 (m, 2H), 1.01-0.95 (m, 12H). **FTIR (KBr)** v_{max}, cm^{-1}: 3450, 3076, 2959, 2927, 1686, 1656, 1606, 1554, 1425-1401, 818-733. **UV-vis** (MeOH): λ_{max}, nm (log *ε*): 306 (3.98), 366 (3.98), 536 (4.25), 564 (4.26). **HR-MS (MALDI-TOF)**: 533.1547 m/z cal. for $C_{29}H_{28}N_2O_4S_2$ (found for $[M+H]^+$ 533.1563).

Synthesis of 1or 3. Methyl 2-(4,4,5,5-tetramethyl-1,3,2-dioxaborolan-2-yl)benzoate or methyl 4-(4,4,5,5-tetramethyl-1,3,2-dioxaborolan-2-yl)benzoate, KOH and THF/H_2O (3: 1) were added to a reaction flask. Subsequently, the reaction was refluxed and stirred for 2h. After that time, the reaction was allowed to reach rt and 2 mL of HCl and H_2O was added and the mixture was extracted with ethyl acetate, dried over anhydrous $MgSO_4$ and the solvent was removed under reduced pressure. The crude product was washed several times with hexane and used in the next step without any other type of purification, obtaining a white solid.
Solid **1**: Yield 60%, **^{1}H NMR** (300 MHz, $CDCl_3$): δ (ppm) 8.06 (d, *J* = 6.0 Hz, 1H), 7.59-7.52 (m, 2H), 7.48-7.45 (m, 1H), 1.42 (s, 12H).
Solid **3:** Yield 90%, **^{1}H NMR** (300 MHz, $CDCl_3$): δ (ppm) 8.10 (d, *J* = 6.0 Hz, 2H), 7.91 (d, *J* = 6.0 Hz, 2H), 1.37 (s, 12H).

1.2.2. Synthesis of PMIDE derivatives

***o*-PMIDE**, ***m*-PMIDE,** and ***p*-PMIDE** were synthesized by a condensation reaction between the perylenemonoanhydridediester (PMADE)[32] and the corresponding substituted amino benzoic acid **4**, **5,** and **6** with yields of 33%, 32%, and 28%, respectively (**Scheme S1b**). Except ***p*-PMIDE**, the others PMIDEs presented good solubility in common organics solvents, such as CH_2Cl_2, $CHCl_3$, and MeOH. In the case of ***p*-PMIDE,** the poor solubility can be explained by the formation of *J*-aggregates. And as it was done for the DPPs, PMIDE derivatives were also properly characterized (**Figures S41-S52**).

Synthesis of PMIDE acid derivatives. Perylenemonoanhydridediester (100 mg, 0.17 mmol), the corresponding aminobenzoic acid (27.7 mg, 0.21 mmol), dimethylaminopyridine (DMAP, 18.33 mg, 0.15 mmol) and imidazole (1.08 g, 15.86 mmol) were added into a round bottom flask. The reaction was heated at 150 ˚C for 5 hours under a nitrogen atmosphere. The mixture was cooled and washed with 2M HCl solution and deionized water. The organic layer was dried over anhydrous Na_2SO_4, filtered and evaporated. Purification was carried out by silica gel column chromatography using toluene/methanol 10:1.

- ***o*-PMIDE**: red solid, 33%. **^{1}H-RMN** (300 MHz, $CDCl_3$) δ(ppm) 8.32 (d, *J* = 7.0 Hz, 1H), 7.76 (t, *J* = 7.1 Hz, 1H), 7.60 (dd, *J* = 13.9, *J* =7.0 Hz, 3H), 7.39 (d, *J* = 7.6 Hz, 1H), 7.04 (bs, 2H), 6.76 (bs, 4H), 4.26 (t, *J* = 7.1 Hz, 4H), 1.88 – 1.74 (m, 4H), 1.51 – 1.30 (m, 12H), 0.93 (t, *J* = 6.9 Hz, 6H). **^{13}C NMR** (75 MHz, $CDCl_3$) δ (ppm) 168.4, 165.37, 164.0, 136.8, 133.5, 130.7, 130.5, 129.8, 129.1, 127.9, 127.2, 126.8, 124.1, 121.5, 120.8, 66.0, 31.6, 28.6, 25.7, 22.7, 14.2. **FTIR (KBr)** v_{max}, cm^{-1} :3420, 2958, 2917, 2860, 1716, 1601, 1368, 1258, 1152, 1062, 809, 751 cm^{-1}. **UV-Vis** ($CHCl_3$) λ_{max}, nm (log *ε*):505 (4.72), 476 (4.60). **HR-MS (MALDI-TOF)**: 697.2676 m/z cal. for $C_{43}H_{39}NO_8$ (found for [M^+] 697.2063).
- ***m*-PMIDE**: red solid, 32%. **^{1}H-RMN** (300 MHz, $CDCl_3$ + 1 drop TFA-d) δ 8.70 (d, *J* = 8.1 Hz, 2H), 8.52 (dd, *J* = 8.2, *J* = 5.3 Hz, 4H), 8.28 (dt, *J* = 7.7, *J* = 1.28 Hz, 1H), 8.15-8.13 (m, 3H), 7.76-7.65 (m, 2H), 4.37 (t, *J* = 6.9 Hz, 4H), 1.81 (q, *J* = 6.95 Hz, 4H), 1.50-140 (m, 4H), 1.38-1.32 (m, 8H), 0.91 (t, *J* = 7.0 Hz, 6H). **^{13}C NMR** (75 MHz, $CDCl_3$) δ 171.5, 160.4, 159.8, 137.0, 133.0, 132.3, 131.8, 131.0, 130.3, 130.2, 123.7, 122., 121.3, 120.2, 116.4, 112.6, 108., 67.06, 9, 31.6, 28.5, 25.7, 22.7, 14.1. **FTIR (KBr)** v_{max}, cm^{-1}: 3420, 3078, 2956, 2927, 2857, 1707, 1667, 1593, 1454, 1416, 1362, 1299, 1267, 1201, 1711, 1104, 1068, 848, 808, 748. **UV-Vis** ($CHCl_3$) λ_{max}, nm (log ε): 508 (4.75), 478 (4.65). **HR-MS (MALDI-TOF)**: 697.2676 m/z cal. for $C_{43}H_{39}NO_8$ (found for [M^+] 697.2073).
- ***p*-PMIDE**: orange solid, 28%. **^{1}H-RMN** (300 MHz, $CDCl_3$ + 1 drop TFA-d) δ 8.76 (d, *J* = 7.9 Hz, 2H), 8.60 (t, *J* = 9.0 Hz 4H), 8.33 (d, *J* = 8.4 Hz, 2H), 8.20 (d, *J* = 7.8 Hz, 2H), 7.52 (d, *J* = 8.4 Hz, 1H), 4.39 (t, *J* = 6.8 Hz, 4H), 1.89 – 1.75 (m, 4H), 1.51 – 1.39 (m, 4H), 1.39 – 130 (m, 8H), 0.91 (t, *J* = 7.0 Hz, 6H). **^{13}C NMR** (75 MHz, $CDCl_3$) δ 171.5, 169.9, 164.7, 160.9, 160.3, 159.7, 159.2, 140.2, 137.1, 133.0, 132.3, 132.1, 131.8, 131.0, 129.8, 129.5, 129.4, 129.1, 129.1, 126.2, 123.8, 122.6, 121.2, 120.3, 116.5, 112.7, 109.0, 67.3, 31.6, 28.5, 25.7, 22.7, 14.0. **FTIR (KBr)** v_{max}, cm^{-1}: 3432, 2962, 2929, 1712, 1671, 1601, 1417, 1356, 1266, 1180, 1119, 809, 645, 617 cm^{-1}. **UV-Vis** ($CHCl_3$) λ_{max}, nm (log ε): 505 (4.75), 478 (4.65). **HR-MS (MALDI-TOF)**: 697.2676 m/z cal. for $C_{43}H_{39}NO_8$ (found for [M^+] 697.2406).

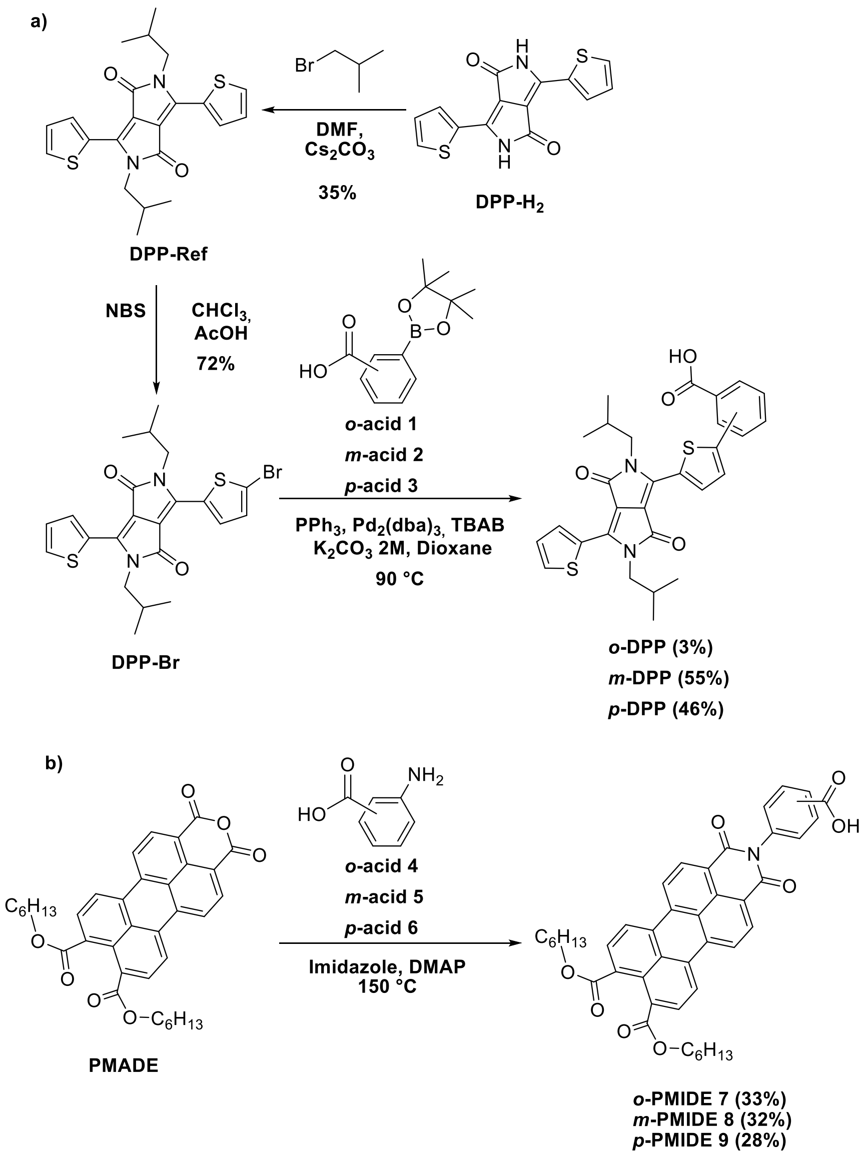

Scheme S1. Synthetic route of **a)** DPP derivatives (***o*-DPP**, ***m*-DPP,** and ***p*-DPP**) and **b)** PMIDE derivatives (***o*-PMIDE**, ***m*-PMIDE,** and ***p*-PMIDE**).

1.3. Optical Properties

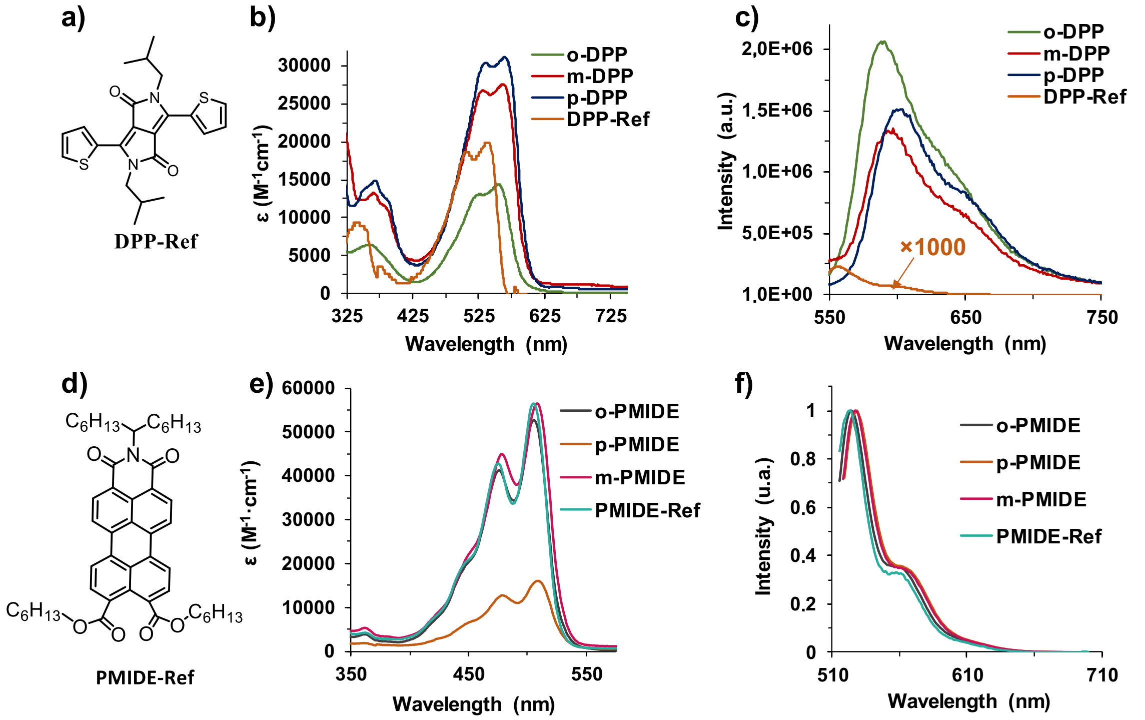

Figure S1. a) DPP-Ref structure. **b**) Room-temperature absorption spectra of ***o*-DPP** (green), ***m*-DPP** (red), ***p*-DPP** (blue), and **DPP-Ref** (orange) carried out in methanol. **c)** Respective fluorescence spectra in methanol following photoexcitation at 550 nm for all compounds. **d) PMIDE-Ref** [33] structure. **e)** Room-temperature absorption spectra of ***o*-PMIDE** (grey), ***m*-PMIDE** (pink), ***p*-PMIDE** (orange)and **PMIDE-Ref** (blue) carried out in $CHCl_3$. **f)** Respective fluorescence spectra in $CHCl_3$ following photoexcitation at 510 nm for all compounds.

1.4. Characterization of nanostructures

1.4.1. CD and optical characterization

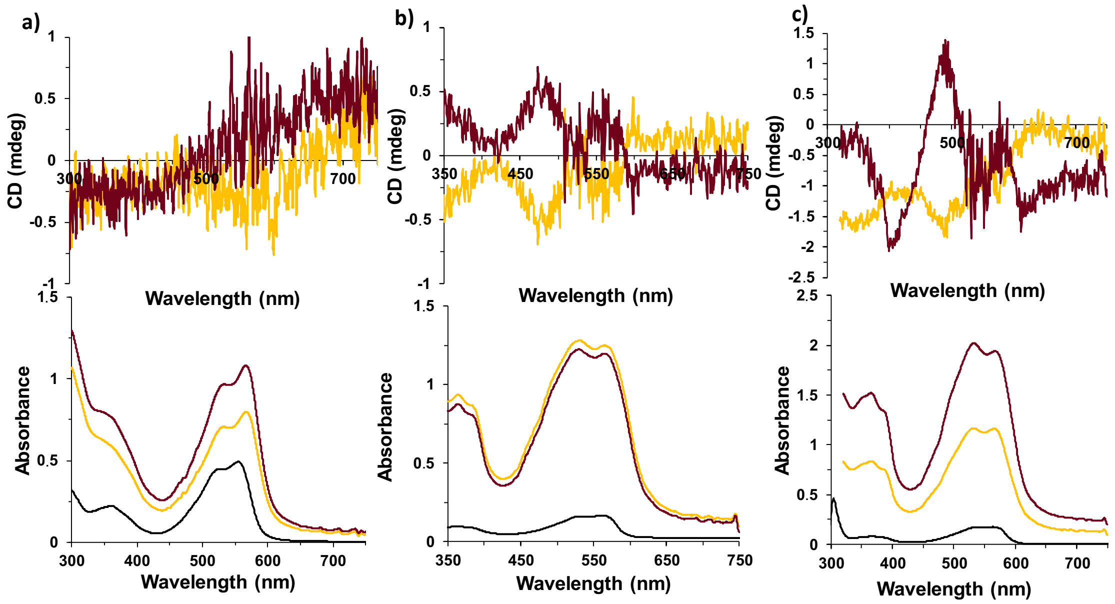

Figure S2. Top corresponds to CD spectra and down to UV-vis spectra in methanol of **a) *o*-DPP** and after the grafting on the surface of the twisted ribbons, **b) *m*-DPP** and after the grafting on the surface of the twisted ribbons and **c) *p*-DPP** and after the grafting on the surface of the twisted ribbons. The yellow line corresponds to the right-handed twisted ribbons, the marrow line to left-handed twisted ribbons and the black to dye-free.

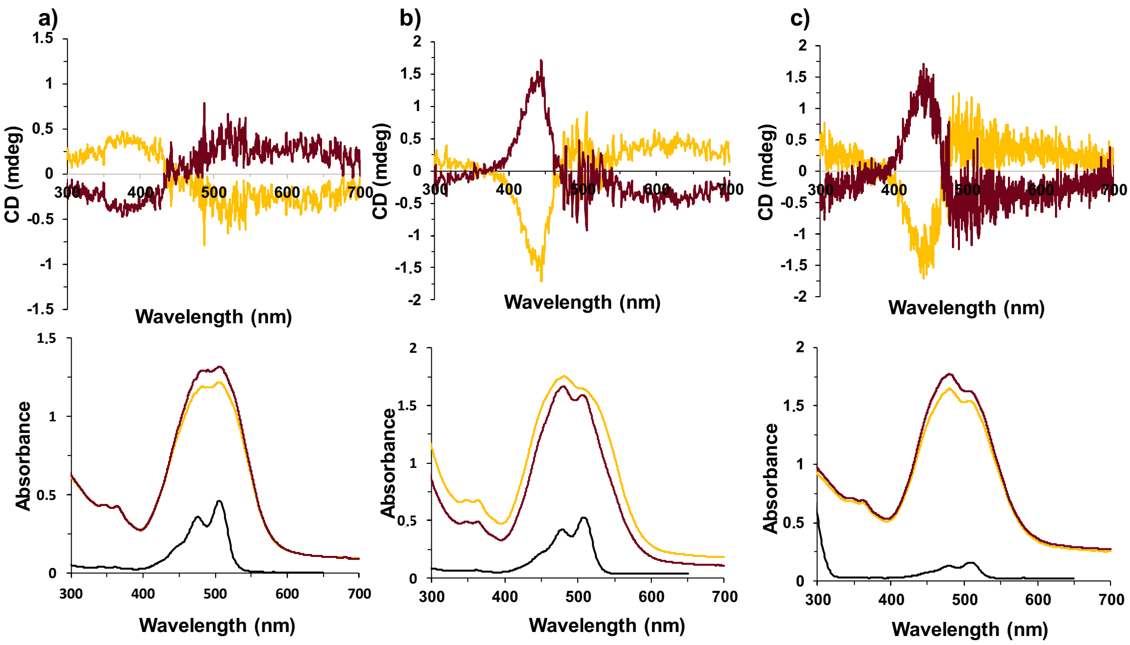

Figure S3. Top corresponds to CD spectra and down to UV-vis spectra in $CHCl_3$ of **a)** ***o*-PMIDE** and after the grafting on the surface of the twisted ribbons, **b)** ***m*-PMIDE** and after the grafting on the surface of the twisted ribbons and **c)** ***p*-PMIDE** and after the grafting on the surface of the twisted ribbons. Yellow line corresponds to the right-handed twisted ribbons, marrow line to left-handed twisted ribbons and black to dye free.

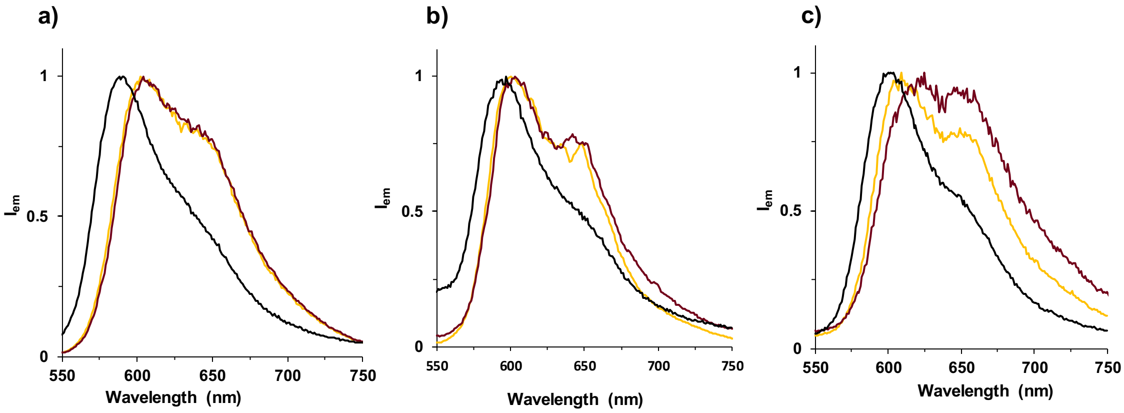

Figure S4. Emission spectra of **a)** ***o*-DPP**, **b)** ***m*-DPP** and **c)** ***p*-DPP** as free dyes (black) and the dyes grafted on the surface of right- (yellow) and left- (marrow) handed twisted ribbons.

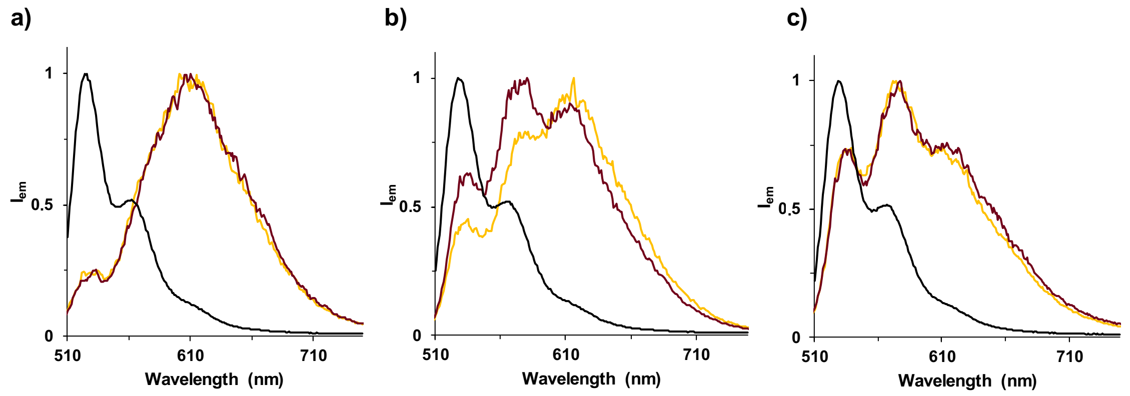

Figure S5. Emission spectra of **a)** ***o*-PMIDE**, **b)** ***m*-PMIDE** and **c)** ***p*-PMIDE** as free dyes (black) and the dyes grafted on the surface of right- (yellow) and left- (marrow) handed twisted ribbons

1.4.2. TEM Characterization

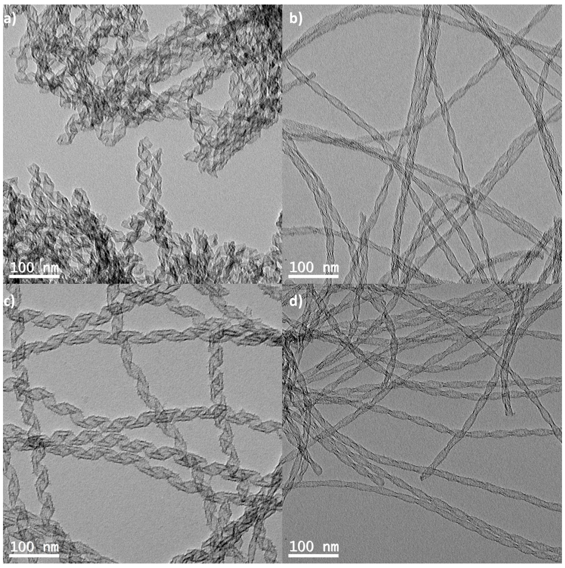

Figure S6. TEM pictures of **a)** and **c)** Right- and left- helical ribbons, respectively**. b)** and **d)** Right- and left- twisted ribbons, respectively.

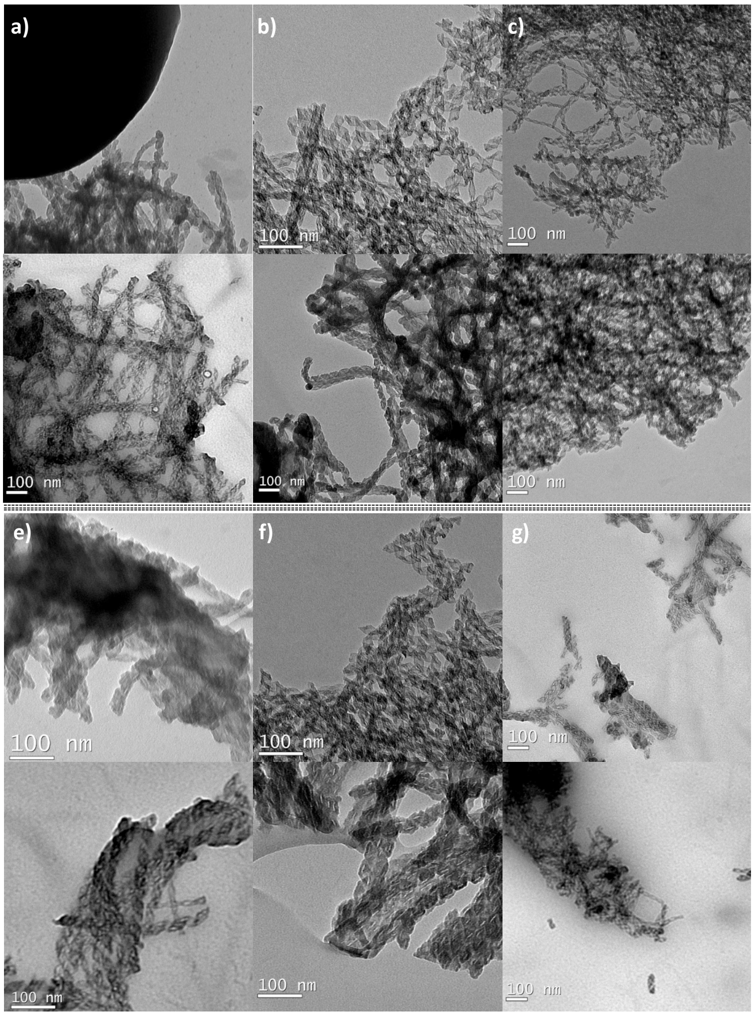

Figure S7.TEM picture of right-(top) and left- (down) handed helical ribbons after grafting of **a)** ***o*-DPP**, **b)** ***m*-DPP**, **c)** ***p*-DPP**, **e)** ***o*-PMIDE**, **b)** ***m*-PMIDE**, **c)** ***p*-PMIDE**.

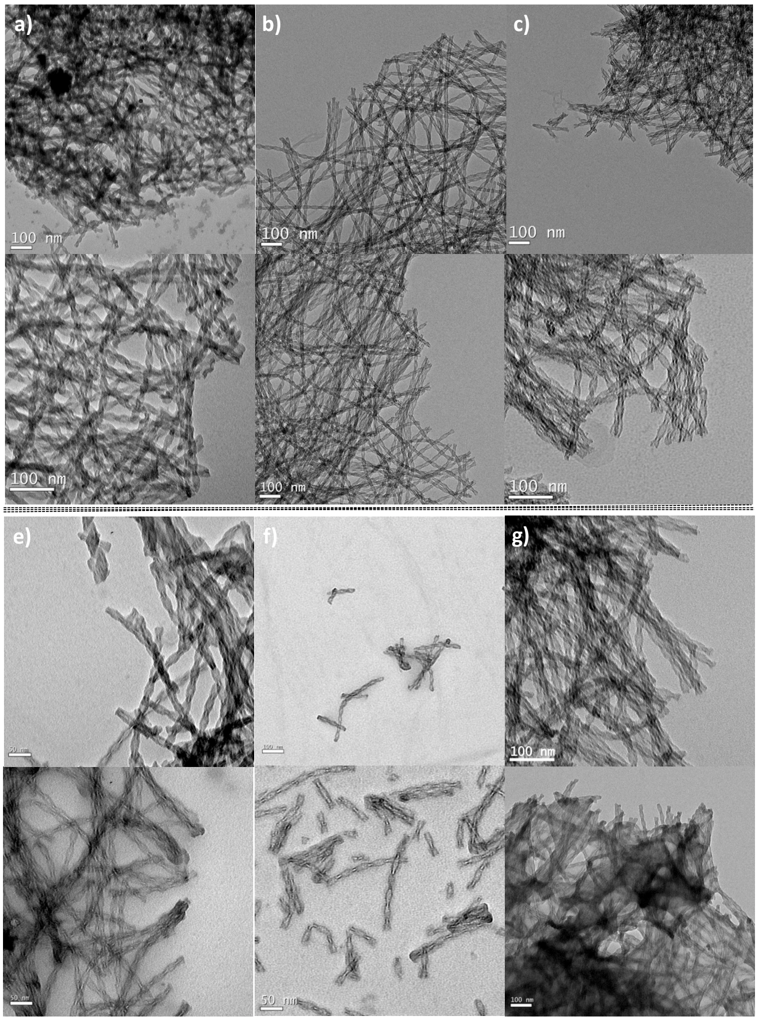

Figure S8. TEM picture of right (top) and left (down) handed twisted ribbons after grafting of **a) *o*-DPP**, **b) *m*-DPP** and **c) *m*-DPP**, **e) *o*-PMIDE**, **b)** ***m*-PMIDE, c) *p*-PMIDE**.

1.4.3. Lifetime Characterization

- **DPP**

a)

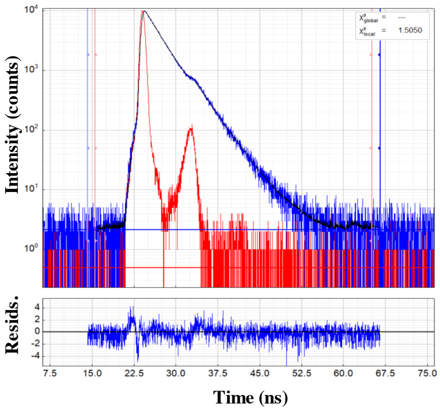

Parameter	Value	Δ	δ
A_1[kCnts/Chnl]	4.93	±0.43	8.7%
τ_1[ns]	2.090	±0.089	4.3%
I_1[kCnts]	412	±47	11%
A_{Rel1}[%]	39.9	±3.4	8.3%
I_{Rel1}[%]	29.5	±3.4	11%
A_2[kCnts/Chnl]	7.43	±0.46	6.1%
τ_2[ns]	3.319	±0.034	1.0%
I_2[kCnts]	986	±50	5.1%
A_{Rel2}[%]	60.2	±3.4	5.5%
I_{Rel2}[%]	70.6	±3.4	4.7%
$Bkgr_{Dec}$[kCnts]	0.0022	±0.0002	5.9%
$Bkgr_{IRF}$[Cnts/Chnl]	0.496	±0.048	9.5%
$Shift_{IRF}$[ps]	227.9	±5.4	2.4%
A_{Scatt}[kCnts]	95.7	±3.9	4.0%
τ_{AvInt}[ns]	2.9568	±0.0061	0.2%
τ_{AvAmp}[ns]	2.829	±0.011	0.4%

b)

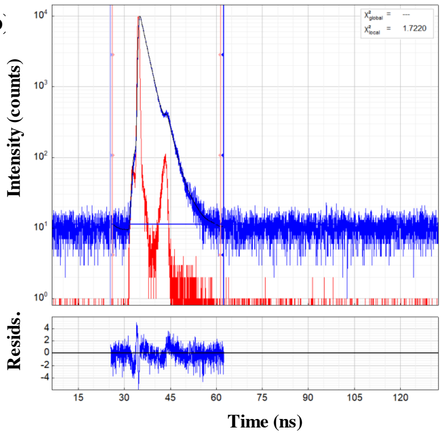

Parameter	Value	Δ	Δ
A_1[kCnts/Chnl]	13.49	±0.19	1.3%
n.o$_1$[ns]	2.099	±0.020	0.9%
I_1[kCnts]	1 133	±25	2.1%
A_{Rel1}[%]	97.4	±1.4	1.4%
I_{Rel1}[%]	95.0	±2,3	2.4%
A_2[kCnts/Chnl]	0.37	±0,20	52%
n.o$_2$[ns]	4.08	±0.52	23F
I_2[kCnts]	61	±27	45%
A_{Rel2}[%]	2.7	±1.4	52%
I_{Rel2}[%]	5.1	±2,3	44%
$Bkgr_{Dec}$[kCnts]	0.0113	±0.0004	2.9%
$Bkgr_{IRF}$[Cnts/Chnl]	0.555	±0.071	23F
$Shift_{IRF}$[ps]	164.2	±2,8	1.7%
Un_{Scatt}[kCnts]	-5.3	±2.0	38%
•$_{AvInt}$[ns]	2.1979	±0.0066	0.3%
•$_{AvAmp}$[ns]	2.1510	±0.0067	0.3%

c)

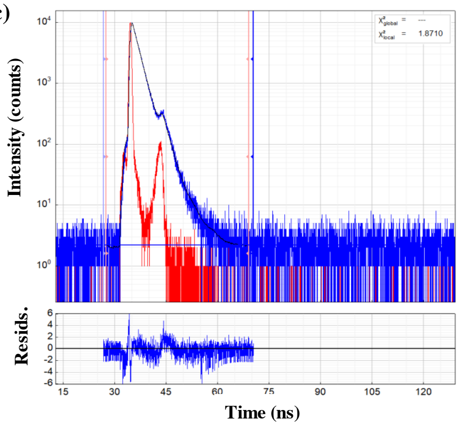

Parameter	Value	Δ	δ
A_1[kCnts/Chnl]	13.85	±0.13	0.9%
τ_1[ns]	1.8509	±0.0085	0.5%
I_1[kCnts]	1 024.8	±9.4	0.9%
A_{Rel1}[%]	96.9	±0.8	0.8%
I_{Rel1}[%]	94.2	±1.1	1.2%
A_2[kCnts/Chnl]	0.45	±0.11	23%
τ_2[ns]	3.55	±0.15	4.1%
I_2[kCnts]	64	±13	19%
A_{Rel2}[%]	3.2	±0.8	24%
I_{Rel2}[%]	5.9	±1.1	19%
$Bkgr_{Dec}$[kCnts]	0.0022	±0.0002	8.4%
$Bkgr_{IRF}$[Cnts/Chnl]	0.121	±0.052	43%
$Shift_{IRF}$[ps]	158.1	±9.0	5.6%
A_{Scatt}[kCnts]	-12.1	±5.7	47%
τ_{AvInt}[ns]	1.9500	±0.0031	0.2%
τ_{AvAmp}[ns]	1.9040	±0.0036	0.2%

Figure S9. Fluorescence time profiles (excitation at 610 nm) of **a) *o*-DPP**, **b) *m*-DPP** and **c) *p*-DPP** in MeOH at rt. The response of the instrument was defined by the black line, the emission decay by the red line and the blue line is the fitting with biexponential decay. The blue line in the residual graphic is the residual data from fitting.

- **DPP- Helical ribbons**

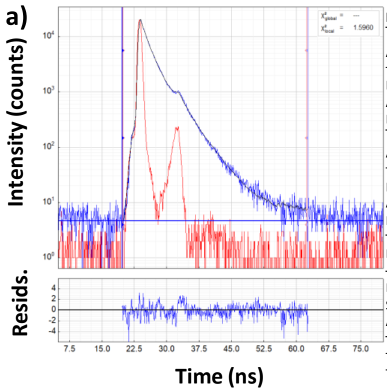

Parameter	Value	Δ	δ
A1[kCnts/Chnl]	6.51	±0.22	3.3%
τ1[ns]	3.785	±0.038	1.0%
I1[kCnts]	246.1	±6.9	2.8%
ARel1[%]	27.4	±1.0	3.6%
IRel1[%]	48.7	±1.5	3.0%
A2[kCnts/Chnl]	17.32	±0.22	1.3%
τ2[ns]	1.501	±0.027	1.8%
I2[kCnts]	259.8	±8.3	3.2%
ARel2[%]	72.7	±1.0	1.4%
IRel2[%]	51.4	±1.5	2.9%
BkgrDec[kCnts]	0.0047	±0.0006	11%
BkgrIRF[Cnts/Chnl]	0.14	±0.16	122%
ShiftIRF[ps]	238.8	±2.6	1.0%
AScatt[kCnts]	72.4	±1.5	2.0%
τAvInt[ns]	2.6113	±0.0094	0.4%
τAvAmp[ns]	2.124	±0.011	0.5%

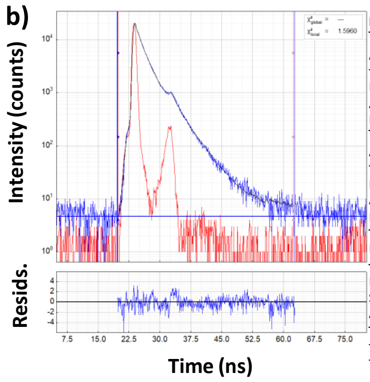

Parameter	Value	Δ	δ
A1[kCnts/Chnl]	6.51	±0.22	3.3%
τ1[ns]	3.785	±0.038	1.0%
I1[kCnts]	246.1	±6.9	2.8%
ARel1[%]	27.4	±1.0	3.6%
IRel1[%]	48.7	±1.5	3.0%
A2[kCnts/Chnl]	17.32	±0.22	1.3%
τ2[ns]	1.501	±0.027	1.8%
I2[kCnts]	259.8	±8.3	3.2%
ARel2[%]	72.7	±1.0	1.4%
IRel2[%]	51.4	±1.5	2.9%
BkgrDec[kCnts]	0.0047	±0.0006	11%
BkgrIRF[Cnts/Chnl]	0.14	±0.16	122%
ShiftIRF[ps]	238.8	±2.6	1.0%
AScatt[kCnts]	72.4	±1.5	2.0%
τAvInt[ns]	2.6113	±0.0094	0.4%
τAvAmp[ns]	2.124	±0.011	0.5%

Figure S10. Fluorescence time profiles (excitation at 610 nm) of ***o*-DPP a)** grafted on right-handed helical ribbons and **b)** grafted on left-handed helical ribbons, in MeOH at rt. The response of the instrument was defined by the black line, the emission decay by the red line and the blue line is the fitting with biexponential decay. The blue line in the residual graphic is the residual data from fitting.

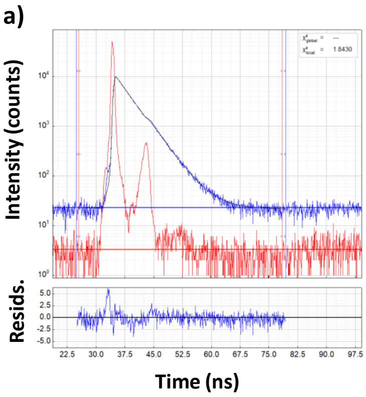

Parameter	Value	Δ	δ
A1[kCnts/Chnl]	13.240	±0.076	0.6%
τ1[ns]	3.931	±0.011	0.3%
I1[kCnts]	520.4	±3.7	0.7%
ARel1[%]	100.0	---	---
IRel1[%]	100.0	---	---
A2[kCnts/Chnl]	- 670	±120	17%
τ2[ns]	0.0570	±0.0034	5.9%
I2[kCnts]	- 384	±61	16%
ARel2[%]	0.0	---	---
IRel2[%]	0.0	---	---
BkgrDec[kCnts]	0.0229	±0.0011	4.5%
BkgrIRF[Cnts/Chnl]	3.27	±0.84	25%
ShiftIRF[ps]	- 116	±32	27%
AScatt[kCnts]	110	±19	17%
τAvInt[ns]	3.931	±0.011	0.3%
τAvAmp[ns]	3.931	±0.011	0.3%

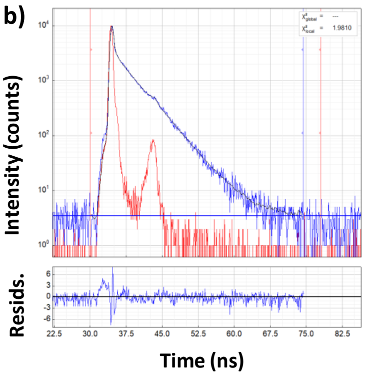

Parameter	Value	Δ	δ
A1[kCnts/Chnl]	3.749	±0.028	0.7%
τ1[ns]	3.996	±0.011	0.3%
I1[kCnts]	149.79	±0.44	0.3%
ARel1[%]	3.9	±0.9	22%
IRel1[%]	50.9	±3.6	6.9%
A2[kCnts/Chnl]	93	±18	19%
τ2[ns]	0.156	±0.017	11%
I2[kCnts]	145	±19	13%
ARel2[%]	96.2	±0.9	0.9%
IRel2[%]	49.2	±3.6	7.2%
BkgrDec[kCnts]	0.0035	±0.0001	2.7%
BkgrIRF[Cnts/Chnl]	0.461	±0.050	11%
ShiftIRF[ps]	-42.0	±5.3	13%
AScatt[kCnts]	-37.8	±8.4	22%
τAvInt[ns]	2.11	±0.14	6.4%
τAvAmp[ns]	0.305	±0.048	16%

Figure S11. Fluorescence time profiles (excitation at 610 nm) of ***m*-DPP a)** grafted on right- handed helical ribbons and **b)** grafted on left-handed helical ribbons, in MeOH at rt. The response of the instrument was defined by the black line, the emission decay by the red line and the blue line is the fitting with biexponential decay. The blue line in the residual graphic is the residual data from fitting.

a)

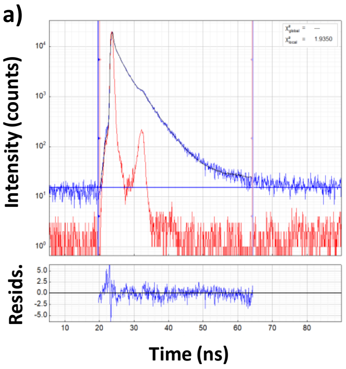

Parameter	Value	Δ	δ
A1[kCnts/Chnl]	7.82	±0.46	5.9%
τ1[ns]	4.350	±0.066	1.5%
I1[kCnts]	341	±16	4.4%
ARel1[%]	50.9	±1.0	2.0%
IRel1[%]	73.5	±2.4	3.2%
A2[kCnts/Chnl]	7.55	±0.25	3.2%
τ2[ns]	1.63	±0.15	9.0%
I2[kCnts]	122.9	±9.3	7.6%
ARel2[%]	49.2	±1.0	2.0%
IRel2[%]	26.6	±2.4	8.9%
BkgrDec[kCnts]	0.0154	±0.0030	19%
BkgrIRF[Cnts/Chnl]	-2.0	±1.1	51%
ShiftIRF[ps]	116.6	±9.9	8.5%
AScatt[kCnts]	104.0	±2.1	2.0%
τAvInt[ns]	3.628	±0.032	0.9%
τAvAmp[ns]	3.014	±0.052	1.7%

b)

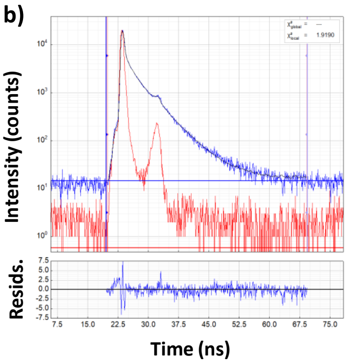

Parameter	Value	Δ	δ
A1[kCnts/Chnl]	3.36	±0.31	9.2%
τ1[ns]	4.80	±0.13	2.6%
I1[kCnts]	162	±12	6.8%
ARel1[%]	0.4	±0.2	42%
IRel1[%]	16.4	±4.2	25%
A2[kCnts/Chnl]	3.91	±0.22	5.5%
τ2[ns]	1.98	±0.18	9.0%
I2[kCnts]	77	±11	14%
ARel2[%]	0.5	±0.2	37%
IRel2[%]	7.9	±1.8	23%
A3[kCnts/Chnl]	910	±630	69%
τ3[ns]	0.083	±0.013	16%
I3[kCnts]	750	±320	43%
ARel3[%]	99.3	±0.4	0.3%
IRel3[%]	75.9	±5.9	7.7%
BkgrDec[kCnts]	0.0146	±0.0020	14%
BkgrIRF[Cnts/Chnl]	0.59	±0.86	148%
ShiftIRF[ps]	63.6	±5.0	7.8%
AScatt[kCnts]	- 234	±96	41%
τAvInt[ns]	1.01	±0.24	24%
τAvAmp[ns]	0.109	±0.024	21%

Figure S12. Fluorescence time profiles (excitation at 610 nm) of ***p*-DPP a)** grafted on right-handed helical ribbons and **b)** grafted on left-handed helical ribbons, in MeOH at rt. The response of the instrument was defined by the black line, the emission decay by the red line and the blue line is the fitting with biexponential decay. The blue line in the residual graphic is the residual data from fitting.

- **DPP- Twisted ribbons**

a)

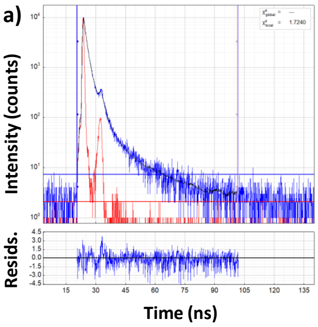

Parameter	Value	Δ	δ
A1[kCnts/Chnl]	11.46	±0.13	1.1%
τ1[ns]	1.176	±0.039	3.3%
I1[kCnts]	134.7	±3.9	2.9%
ARel1[%]	85.2	±1.7	2.0%
IRel1[%]	64.0	±2.5	3.8%
A2[kCnts/Chnl]	0.0490	±0.0082	17%
τ2[ns]	18.1	±1.8	9.7%
I2[kCnts]	8.84	±0.55	6.1%
ARel2[%]	0.4	±0.1	17%
IRel2[%]	4.2	±0.3	5.9%
A3[kCnts/Chnl]	1.95	±0.27	14%
τ3[ns]	3.46	±0.23	6.4%
I3[kCnts]	67.3	±5.6	8.3%
ARel3[%]	14.5	±1.8	12%
IRel3[%]	32.0	±2.4	7.4%
BkgrDec[kCnts]	0.0074	±0.0024	33%
BkgrIRF[Cnts/Chnl]	2.08	±0.67	32%
ShiftIRF[ps]	74.0	±7.6	10%
AScatt[kCnts]	27.7	±2.1	7.3%
τAvInt[ns]	2.609	±0.048	1.8%
τAvAmp[ns]	1.567	±0.022	1.4%

b)

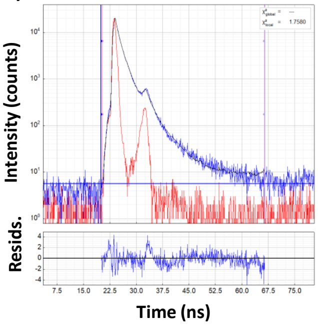

Parameter	Value	Δ	δ
A1[kCnts/Chnl]	17.88	±0.47	2.6%
τ1[ns]	1.432	±0.026	1.8%
I1[kCnts]	255.9	±6.4	2.5%
ARel1[%]	31	±15	48%
IRel1[%]	61.4	±6.7	11%
A2[kCnts/Chnl]	1.94	±0.17	8.5%
τ2[ns]	4.15	±0.11	2.7%
I2[kCnts]	80.2	±5.1	6.3%
ARel2[%]	3.4	±1.9	57%
IRel2[%]	19.3	±3.0	15%
A3[kCnts/Chnl]	39	±55	142%
τ3[ns]	0.212	±0.073	34%
I3[kCnts]	81	±48	59%
ARel3[%]	66	±17	25%
IRel3[%]	19.4	±8.0	41%
BkgrDec[kCnts]	0.0057	±0.0008	14%
BkgrIRF[Cnts/Chnl]	-0.43	±0.39	93%
ShiftIRF[ps]	185	±11	5.8%
AScatt[kCnts]	44	±35	78%
τAvInt[ns]	1.72	±0.18	10%
τAvAmp[ns]	0.72	±0.31	42%

Figure S13. Fluorescence time profiles (excitation at 610 nm) of ***o*-DPP a)** grafted on right-handed *twisted* ribbons and **b)** grafted on left-handed *twisted* ribbons, in MeOH at rt. The response of the instrument was defined by the black line, the emission decay by the red line and the blue line is the fitting with multiexponential decay. The blue line in the residual graphic is the residual data from fitting.

a)

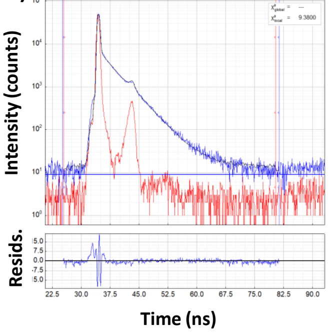

Parameter	Value	Δ	δ
A1[kCnts/Chnl]	1 280	±260	20%
τ1[ns]	0.146	±0.017	11%
I1[kCnts]	1 870	±400	21%
ARel1[%]	99.5	±0.2	0.1%
IRel1[%]	85.9	±2.2	2.5%
A2[kCnts/Chnl]	7.396	±0.088	1.2%
τ2[ns]	4.151	±0.026	0.6%
I2[kCnts]	307.0	±1.6	0.5%
ARel2[%]	0.6	±0.2	17%
IRel2[%]	14.2	±2.2	15%
A3[kCnts/Chnl]	- 450	±130	27%
τ3[ns]	0.186	±0.016	8.5%
I3[kCnts]	- 850	±280	33%
ARel3[%]	0.0	---	---
IRel3[%]	0.0	---	---
BkgrDec[kCnts]	0.0091	±0.0027	29%
BkgrIRF[Cnts/Chnl]	-0.7	±1.8	284%
ShiftIRF[ps]	-46.5	±3.4	7.3%
AScatt[kCnts]	- 278	±69	25%
τAvInt[ns]	0.72	±0.11	15%
τAvAmp[ns]	0.169	±0.021	12%

b)

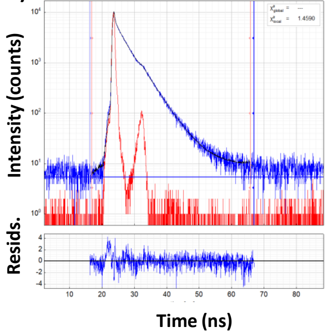

Parameter	Value	Δ	δ
A1[kCnts/Chnl]	2.159	±0.091	4.2%
τ1[ns]	1.48	±0.12	8.1%
I1[kCnts]	63.7	±8.3	13%
ARel1[%]	26.7	±1.2	4.2%
IRel1[%]	11.2	±1.5	13%
A2[kCnts/Chnl]	5.93	±0.17	2.8%
τ2[ns]	4.292	±0.038	0.9%
I2[kCnts]	508.7	±9.6	1.9%
ARel2[%]	73.4	±1.2	1.5%
IRel2[%]	88.9	±1.5	1.7%
BkgrDec[kCnts]	0.0055	±0.0003	4.5%
BkgrIRF[Cnts/Chnl]	-0.791	±0.072	9.1%
ShiftIRF[ps]	-44.2	±4.4	9.8%
AScatt[kCnts]	96.8	±2.0	2.0%
τAvInt[ns]	3.978	±0.011	0.3%
τAvAmp[ns]	3.540	±0.035	1.0%

Figure S14. Fluorescence time profiles (excitation at 610 nm) of ***m*-DPP a)** grafted on right-handed *twisted* ribbons and **b)** grafted on left-handed *twisted* ribbons, in MeOH at rt. The response of the instrument was defined by the black line, the emission decay by the red line and the blue line is the fitting with multiexponential decay. The blue line in the residual graphic is the residual data from fitting.

a)

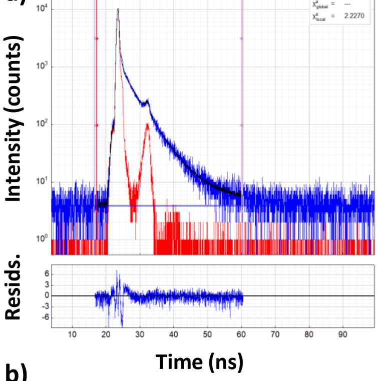

Parameter	Value	Δ	δ
A1[kCnts/Chnl]	1.113	±0.015	1.3%
τ1[ns]	4.028	±0.076	1.9%
I1[kCnts]	179.3	±3.1	1.7%
ARel1[%]	97.3	±1.1	1.1%
IRel1[%]	92.1	±1.9	2.0%
A2[kCnts/Chnl]	0.032	±0.013	40%
τ2[ns]	12.2	±2.0	16%
I2[kCnts]	15.5	±3.8	24%
ARel2[%]	2.8	±1.1	38%
IRel2[%]	8.0	±1.9	24%
BkgrDec[kCnts]	0.0039	±0.0003	6.3%
BkgrIRF[Cnts/Chnl]	0.20	±0.20	99%
ShiftIRF[ps]	-56.0	±1.5	2.5%
AScatt[kCnts]	332.1	±1.2	0.4%
τAvInt[ns]	4.673	±0.087	1.8%
τAvAmp[ns]	4.253	±0.052	1.2%

b)

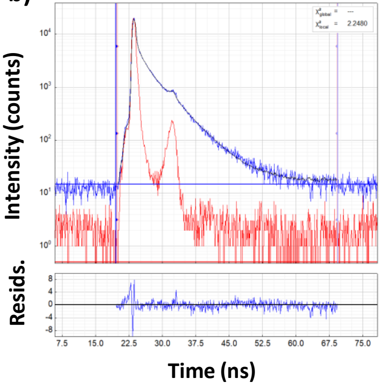

Parameter	Value	Δ	δ
A1[kCnts/Chnl]	3.81	±0.30	7.8%
τ1[ns]	4.60	±0.11	2.4%
I1[kCnts]	175.2	±9.3	5.3%
ARel1[%]	49.0	±2.1	4.3%
IRel1[%]	74.1	±3.1	4.2%
A2[kCnts/Chnl]	3.97	±0.19	4.8%
τ2[ns]	1.55	±0.23	14%
I2[kCnts]	61.5	±6.8	11%
ARel2[%]	51.1	±2.1	4.1%
IRel2[%]	26.0	±3.1	12%
BkgrDec[kCnts]	0.0149	±0.0009	5.6%
BkgrIRF[Cnts/Chnl]	0.50	±0.68	135%
ShiftIRF[ps]	155.5	±6.3	4.0%
AScatt[kCnts]	150.5	±7.2	4.8%
τAvInt[ns]	3.808	±0.057	1.5%
τAvAmp[ns]	3.05	±0.17	5.4%

Figure S15. Fluorescence time profiles (excitation at 610 nm) of ***p*-DPP a)** grafted on right-handed *twisted* ribbons and **b)** grafted on left-handed *twisted* ribbons, in MeOH at rt. The response of the instrument was defined by the black line, the emission decay by the red line and the blue line is the fitting with biexponential decay. The blue line in the residual graphic is the residual data from fitting.

- **PMIDE**

a)

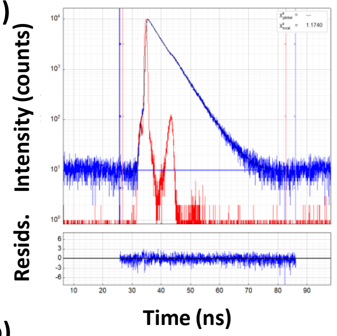

Parameter	Value	Δ	δ
A1[kCnts/Chnl]	12.253	±0.023	0.2%
τ1[ns]	4.6677	±0.0030	0.1%
I1[kCnts]	2 287.8	±3.3	0.1%
ARel1[%]	100.0	---	---
IRel1[%]	100.0	---	---
BkgrDec[kCnts]	0.0098	±0.0003	2.1%
BkgrIRF[Cnts/Chnl]	0.291	±0.034	11%
ShiftIRF[ps]	151.9	±7.0	4.6%
AScatt[kCnts]	-41.7	±2.3	5.5%
τAvInt[ns]	4.6677	±0.0030	0.1%
τAvAmp[ns]	4.6677	±0.0030	0.1%

b)

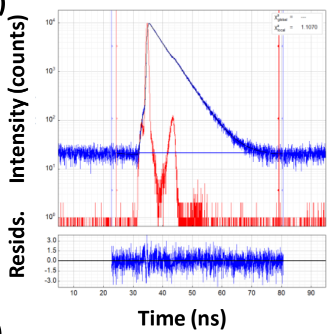

Parameter	Value	Δ	δ
A1[kCnts/Chnl]	12.314	±0.012	0.1%
τ1[ns]	4.5967	±0.0039	0.1%
I1[kCnts]	2 264.0	±4.0	0.2%
ARel1[%]	100.0	---	---
IRel1[%]	100.0	---	---
BkgrDec[kCnts]	0.0211	±0.0003	1.4%
BkgrIRF[Cnts/Chnl]	0.366	±0.036	9.6%
ShiftIRF[ps]	159.5	±8.2	5.1%
AScatt[kCnts]	-37.7	±4.2	11%
τAvInt[ns]	4.5967	±0.0039	0.1%
τAvAmp[ns]	4.5967	±0.0039	0.1%

c)

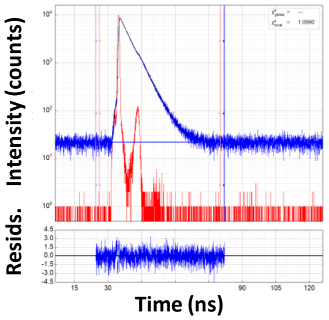

Parameter	Value	Δ	δ
A1[kCnts/Chnl]	10.830	±0.011	0.1%
τ1[ns]	4.3337	±0.0033	0.1%
I1[kCnts]	1 877.2	±2.0	0.1%
ARel1[%]	100.0	---	---
IRel1[%]	100.0	---	---
BkgrDec[kCnts]	0.0219	±0.0003	1.1%
BkgrIRF[Cnts/Chnl]	0.132	±0.064	48%
ShiftIRF[ps]	114.7	±5.2	4.5%
AScatt[kCnts]	-46.8	±1.9	4.0%
τAvInt[ns]	4.3337	±0.0033	0.1%
τAvAmp[ns]	4.3337	±0.0033	0.1%

Figure S16. Fluorescence time profiles (excitation at 530 nm) of **a) *o*-PMIDE**, **b) *m*-PMIDE** and ***c)* *p*-PMIDE** in $CHCl_3$ at rt. The response of the instrument was defined by the black line, the emission decay by the red *line and the blue line is the fitting with monoexponential decay. The blue line in the residual graphic is the residual data from fitting.*

- **PMIDE- Helical ribbons**

a)

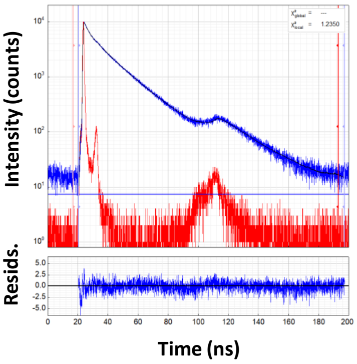

Parameter	Value	Δ	δ
A1[kCnts/Chnl]	2.25	±0.36	16%
τ1[ns]	25.34	±0.98	3.9%
I1[kCnts]	1 140	±140	12%
ARel1[%]	18.4	±3.0	16%
IRel1[%]	41.4	±5.0	12%
A2[kCnts/Chnl]	5.22	±0.27	5.1%
τ2[ns]	12.94	±0.66	5.1%
I2[kCnts]	1 350	±130	9.1%
ARel2[%]	42.7	±1.9	4.4%
IRel2[%]	49.2	±4.5	9.1%
A3[kCnts/Chnl]	4.79	±0.16	3.3%
τ3[ns]	2.74	±0.15	5.2%
I3[kCnts]	262	±14	5.3%
ARel3[%]	39.1	±1.4	3.4%
IRel3[%]	9.6	±0.6	5.5%
BkgrDec[kCnts]	0.0074	±0.0036	49%
BkgrIRF[Cnts/Chnl]	0.79	±0.21	26%
ShiftIRF[ps]	128.5	±9.9	7.7%
AScatt[kCnts]	34.2	±3.8	11%
τAvInt[ns]	17.097	±0.047	0.3%
τAvAmp[ns]	11.23	±0.13	1.2%

b)

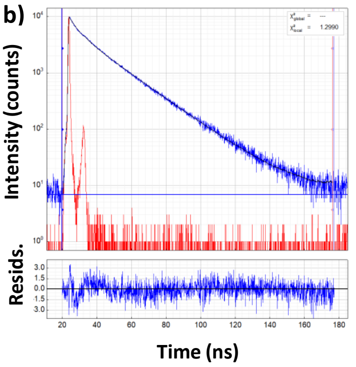

Parameter	Value	Δ	δ
A1[kCnts/Chnl]	4.577	±0.091	2.0%
τ1[ns]	4.69	±0.21	4.5%
I1[kCnts]	214.4	±4.7	2.2%
ARel1[%]	43.2	±0.5	0.9%
IRel1[%]	15.4	±0.4	2.4%
A2[kCnts/Chnl]	6.021	±0.054	0.9%
τ2[ns]	19.596	±0.081	0.4%
I2[kCnts]	1 179.8	±7.4	0.6%
ARel2[%]	56.9	±0.5	0.7%
IRel2[%]	84.7	±0.4	0.4%
BkgrDec[kCnts]	0.0069	±0.0012	17%
BkgrIRF[Cnts/Chnl]	0.271	±0.061	22%
ShiftIRF[ps]	181.1	±4.0	2.2%
AScatt[kCnts]	22.0	±1.3	5.8%
τAvInt[ns]	17.303	±0.037	0.2%
τAvAmp[ns]	13.16	±0.16	1.2%

Figure S17. Fluorescence time profiles (excitation at 650 nm) of ***o*-PMIDE a)** grafted on right-handed helical ribbons and **b)** grafted on left-handed helical ribbons, in $CHCl_3$ at rt. The response of the instrument was defined by the black line, the emission decay by the red line and the blue line is the fitting with multiexponential decay. The blue line in the residual graphic is the residual data from fitting.

a)

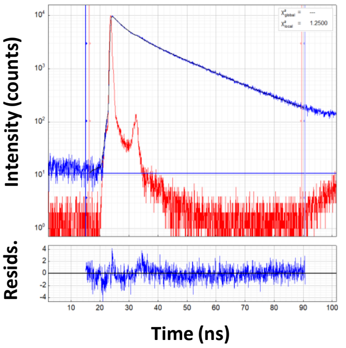

Parameter	Value	Δ	δ
A1[kCnts/Chnl]	6.142	±0.031	0.5%
τ1[ns]	3.820	±0.025	0.6%
I1[kCnts]	469.1	±7.1	1.5%
ARel1[%]	51.7	±0.3	0.4%
IRel1[%]	18.3	±0.3	1.3%
A2[kCnts/Chnl]	5.758	±0.034	0.6%
τ2[ns]	18.287	±0.071	0.4%
I2[kCnts]	2 105.7	±4.8	0.2%
ARel2[%]	48.4	±0.3	0.4%
IRel2[%]	81.8	±0.3	0.3%
BkgrDec[kCnts]	0.0110	±0.0007	5.6%
BkgrIRF[Cnts/Chnl]	0.22	±0.20	93%
ShiftIRF[ps]	166.7	±5.3	3.2%
AScatt[kCnts]	35.3	±1.8	4.9%
τAvInt[ns]	15.651	±0.044	0.3%
τAvAmp[ns]	10.820	±0.020	0.2%

b)

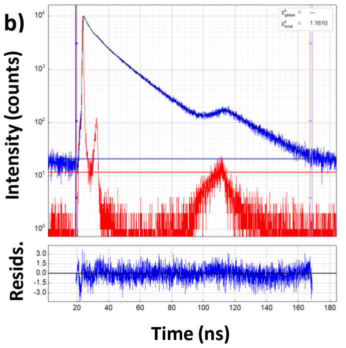

Parameter	Value	Δ	δ
A1[kCnts/Chnl]	5.569	±0.037	0.7%
τ1[ns]	3.821	±0.079	2.1%
I1[kCnts]	426	±12	2.8%
ARel1[%]	47.1	±0.4	0.7%
IRel1[%]	8.1	±2.6	32%
A2[kCnts/Chnl]	0.248	±0.015	6.0%
τ2[ns]	580	±420	71%
I2[kCnts]	2 900	±2 600	91%
ARel2[%]	2.1	±0.2	6.2%
IRel2[%]	54	±16	28%
A3[kCnts/Chnl]	6.011	±0.058	1.0%
τ3[ns]	16.90	±0.11	0.6%
I3[kCnts]	2 031.4	±5.6	0.3%
ARel3[%]	50.9	±0.5	0.8%
IRel3[%]	39	±13	33%
BkgrDec[kCnts]	0.0210	±0.0014	6.2%
BkgrIRF[Cnts/Chnl]	11.8	±1.9	15%
ShiftIRF[ps]	181.2	±4.7	2.6%
AScatt[kCnts]	26.7	±1.8	6.7%
τAvInt[ns]	320	±370	117%
τAvAmp[ns]	23	±11	48%

Figure S18. Fluorescence time profiles (excitation at 650 nm) of ***m*-PMIDE a)** grafted on right-handed helical ribbons and **b)** grafted on left-handed helical ribbons, in $CHCl_3$ at rt. The response of the instrument was defined by the black line, the emission decay by the red line and the blue line is the fitting with multiexponential decay. The blue line in the residual graphic is the residual data from fitting.

a)

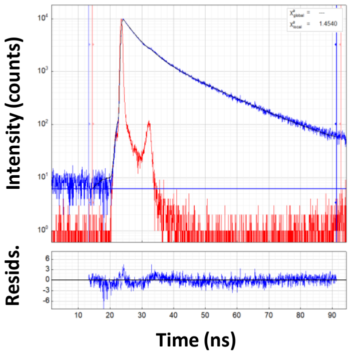

Parameter	Value	Δ	δ
A1[kCnts/Chnl]	2.939	±0.040	1.3%
τ1[ns]	15.85	±0.13	0.8%
I1[kCnts]	931.4	±6.7	0.7%
ARel1[%]	24.5	±0.4	1.5%
IRel1[%]	56.9	±0.5	0.8%
A2[kCnts/Chnl]	9.059	±0.079	0.9%
τ2[ns]	3.904	±0.034	0.9%
I2[kCnts]	707.3	±10.0	1.4%
ARel2[%]	75.6	±0.4	0.5%
IRel2[%]	43.2	±0.5	1.1%
BkgrDec[kCnts]	0.0062	±0.0004	5.1%
BkgrIRF[Cnts/Chnl]	-0.23	±0.13	55%
ShiftIRF[ps]	67	±13	18%
AScatt[kCnts]	14.9	±3.6	24%
τAvInt[ns]	10.692	±0.037	0.3%
τAvAmp[ns]	6.829	±0.028	0.4%

b)

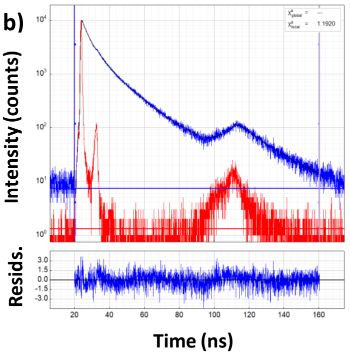

Parameter	Value	Δ	δ
A1[kCnts/Chnl]	7.785	±0.064	0.8%
τ1[ns]	3.568	±0.042	1.2%
I1[kCnts]	555.4	±9.2	1.6%
ARel1[%]	64.9	±0.5	0.7%
IRel1[%]	31.6	±0.4	1.1%
A2[kCnts/Chnl]	0.323	±0.061	19%
τ2[ns]	37.5	±5.3	14%
I2[kCnts]	243	±20	8.2%
ARel2[%]	2.7	±0.6	19%
IRel2[%]	13.8	±1.3	8.8%
A3[kCnts/Chnl]	3.898	±0.053	1.3%
τ3[ns]	12.37	±0.31	2.4%
I3[kCnts]	964	±21	2.2%
ARel3[%]	32.5	±0.3	0.8%
IRel3[%]	54.8	±0.9	1.6%
BkgrDec[kCnts]	0.0074	±0.0006	8.0%
BkgrIRF[Cnts/Chnl]	1.30	±0.29	22%
ShiftIRF[ps]	126.0	±6.6	5.2%
AScatt[kCnts]	36.4	±1.8	4.8%
τAvInt[ns]	13.05	±0.47	3.6%
τAvAmp[ns]	7.337	±0.048	0.6%

Figure S19. Fluorescence time profiles (excitation at 650 nm) of ***p*-PMIDE a)** grafted on right-handed helical ribbons and **b)** grafted on left-handed helical ribbons, in $CHCl_3$ at rt. The response of the instrument was defined by the black line, the emission decay by the red line and the blue line is the fitting with multiexponential decay. The blue line in the residual graphic is the residual data from fitting.

- ## PMIDE- Twisted ribbons

a)

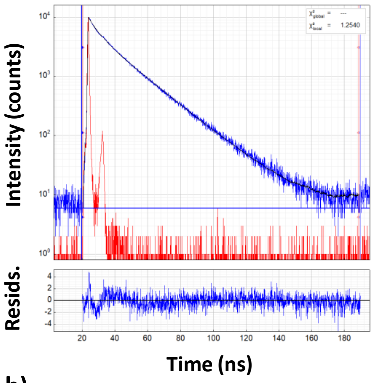

Parameter	Value	Δ	δ
A1[kCnts/Chnl]	6.016	±0.030	0.5%
τ1[ns]	19.268	±0.041	0.2%
I1[kCnts]	1 159.1	±3.4	0.3%
ARel1[%]	56.9	±0.3	0.5%
IRel1[%]	83.3	±0.3	0.3%
A2[kCnts/Chnl]	4.558	±0.050	1.1%
τ2[ns]	5.101	±0.090	1.8%
I2[kCnts]	232.5	±4.2	1.8%
ARel2[%]	43.2	±0.3	0.7%
IRel2[%]	16.8	±0.3	1.7%
BkgrDec[kCnts]	0.0060	±0.0008	13%
BkgrIRF[Cnts/Chnl]	0.384	±0.046	12%
ShiftIRF[ps]	193.9	±2.9	1.5%
AScatt[kCnts]	24.72	±0.98	4.0%
τAvInt[ns]	16.902	±0.018	0.1%
τAvAmp[ns]	13.161	±0.052	0.4%

b)

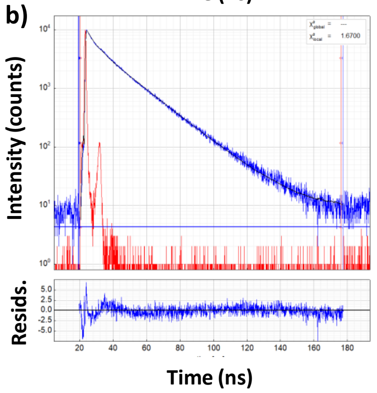

Parameter	Value	Δ	δ
A1[kCnts/Chnl]	5.410	±0.099	1.8%
τ1[ns]	4.41	±0.18	3.9%
I1[kCnts]	238.4	±8.4	3.5%
ARel1[%]	46.0	±0.8	1.7%
IRel1[%]	16.4	±0.6	3.6%
A2[kCnts/Chnl]	6.357	±0.076	1.2%
τ2[ns]	19.21	±0.13	0.6%
I2[kCnts]	1 220.9	±9.0	0.7%
ARel2[%]	54.1	±0.8	1.4%
IRel2[%]	83.7	±0.6	0.7%
BkgrDec[kCnts]	0.0044	±0.0020	45%
BkgrIRF[Cnts/Chnl]	0.08	±0.15	190%
ShiftIRF[ps]	- 208.0	±8.0	3.8%
AScatt[kCnts]	-26.64	±0.57	2.1%
τAvInt[ns]	16.790	±0.041	0.2%
τAvAmp[ns]	12.41	±0.18	1.4%

Figure S20. Fluorescence time profiles (excitation at 650 nm) of ***o*-PMIDE a)** grafted on right-handed *twisted* ribbons and **b)** grafted on left-handed *twisted* ribbons, in $CHCl_3$ at rt. The response of the instrument was defined by the black line, the emission decay by the red line and the blue line is the fitting with biexponential decay. The blue line in the residual graphic is the residual data from fitting.

a)

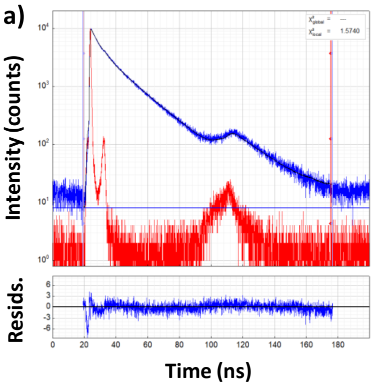

Parameter	Value	Δ	δ
A1[kCnts/Chnl]	5.053	±0.025	0.5%
τ1[ns]	18.685	±0.039	0.2%
I1[kCnts]	1 888.3	±4.3	0.2%
ARel1[%]	43.6	±0.2	0.3%
IRel1[%]	75.3	±0.3	0.3%
A2[kCnts/Chnl]	6.558	±0.034	0.5%
τ2[ns]	4.733	±0.045	0.9%
I2[kCnts]	620.7	±5.2	0.8%
ARel2[%]	56.5	±0.2	0.3%
IRel2[%]	24.8	±0.3	0.8%
BkgrDec[kCnts]	0.0082	±0.0009	11%
BkgrIRF[Cnts/Chnl]	0.735	±0.072	9.7%
ShiftIRF[ps]	213.1	±5.2	2.4%
AScatt[kCnts]	46.9	±2.2	4.7%
τAvInt[ns]	15.234	±0.014	0.1%
τAvAmp[ns]	10.805	±0.035	0.3%

b)

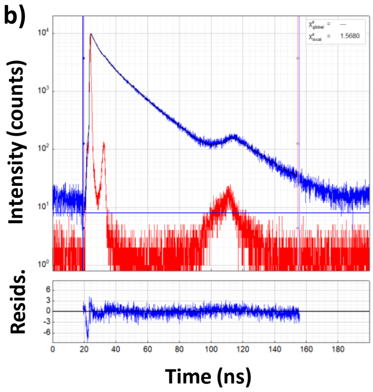

Parameter	Value	Δ	δ
A1[kCnts/Chnl]	5.190	±0.037	0.7%
τ1[ns]	18.342	±0.069	0.4%
I1[kCnts]	1 903.8	±6.9	0.4%
ARel1[%]	44.6	±0.4	0.7%
IRel1[%]	76.4	±0.3	0.3%
A2[kCnts/Chnl]	6.459	±0.060	0.9%
τ2[ns]	4.572	±0.047	1.0%
I2[kCnts]	590.6	±7.0	1.2%
ARel2[%]	55.5	±0.4	0.6%
IRel2[%]	23.7	±0.3	1.0%
BkgrDec[kCnts]	0.0080	±0.0010	12%
BkgrIRF[Cnts/Chnl]	0.407	±0.081	20%
ShiftIRF[ps]	210	±13	6.2%
AScatt[kCnts]	45.1	±4.3	9.3%
τAvInt[ns]	15.082	±0.033	0.2%
τAvAmp[ns]	10.707	±0.051	0.5%

Figure S21. Fluorescence time profiles (excitation at 650 nm) of ***m*-PMIDE a)** grafted on right-handed *twisted* ribbons and **b)** grafted on left-handed *twisted* ribbons, in $CHCl_3$ at rt. The response of the instrument was defined by the black line, the emission decay by the red line and the blue line is the fitting with biexponential decay. The blue line in the residual graphic is the residual data from fitting.

a)

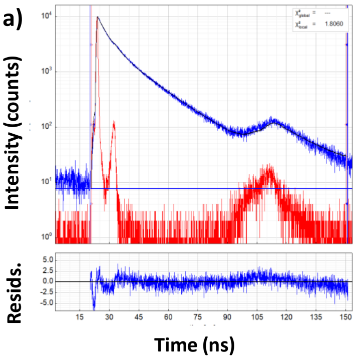

Parameter	Value	Δ	δ
A1[kCnts/Chnl]	7.662	±0.052	0.7%
τ1[ns]	4.520	±0.059	1.3%
I1[kCnts]	693	±12	1.6%
ARel1[%]	68.2	±0.5	0.6%
IRel1[%]	36.3	±0.6	1.6%
A2[kCnts/Chnl]	3.588	±0.057	1.6%
τ2[ns]	16.99	±0.12	0.7%
I2[kCnts]	1 219	±12	0.9%
ARel2[%]	31.9	±0.5	1.3%
IRel2[%]	63.8	±0.6	0.9%
BkgrDec[kCnts]	0.0076	±0.0008	10%
BkgrIRF[Cnts/Chnl]	-0.123	±0.086	70%
ShiftIRF[ps]	197.7	±3.5	1.7%
AScatt[kCnts]	54.4	±1.6	2.9%
τAvInt[ns]	12.466	±0.039	0.3%
τAvAmp[ns]	8.494	±0.034	0.4%

b)

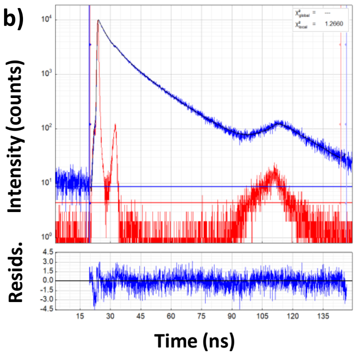

Parameter	Value	Δ	δ
A1[kCnts/Chnl]	0.178	±0.012	6.4%
τ1[ns]	89	±15	16%
I1[kCnts]	314	±28	8.6%
ARel1[%]	1.6	±0.1	6.2%
IRel1[%]	15.1	±1.1	6.8%
A2[kCnts/Chnl]	4.555	±0.076	1.7%
τ2[ns]	13.86	±0.20	1.4%
I2[kCnts]	1 261.8	±7.3	0.6%
ARel2[%]	39.6	±0.5	1.1%
IRel2[%]	60.6	±1.3	2.0%
A3[kCnts/Chnl]	6.770	±0.051	0.7%
τ3[ns]	3.751	±0.071	1.9%
I3[kCnts]	508	±13	2.4%
ARel3[%]	58.9	±0.6	0.8%
IRel3[%]	24.4	±0.6	2.1%
BkgrDec[kCnts]	0.0087	±0.0004	3.9%
BkgrIRF[Cnts/Chnl]	4.35	±0.54	12%
ShiftIRF[ps]	169.4	±2.6	1.5%
AScatt[kCnts]	50.68	±0.76	1.5%
τAvInt[ns]	22.6	±3.0	13%
τAvAmp[ns]	9.06	±0.21	2.3%

Figure S22. Fluorescence time profiles (excitation at 650 nm) of ***p*-PMIDE a)** grafted on right-handed *twisted* ribbons and **b)** grafted on left-handed *twisted* ribbons, in $CHCl_3$ at rt. The response of the instrument was defined by the black line, the emission decay by the red line and the blue line is the fitting with multiexponential decay. The blue line in the residual graphic is the residual data from fitting.

1.5. Structural Characterization

1.5.1. Characterization of the DPP derivatives

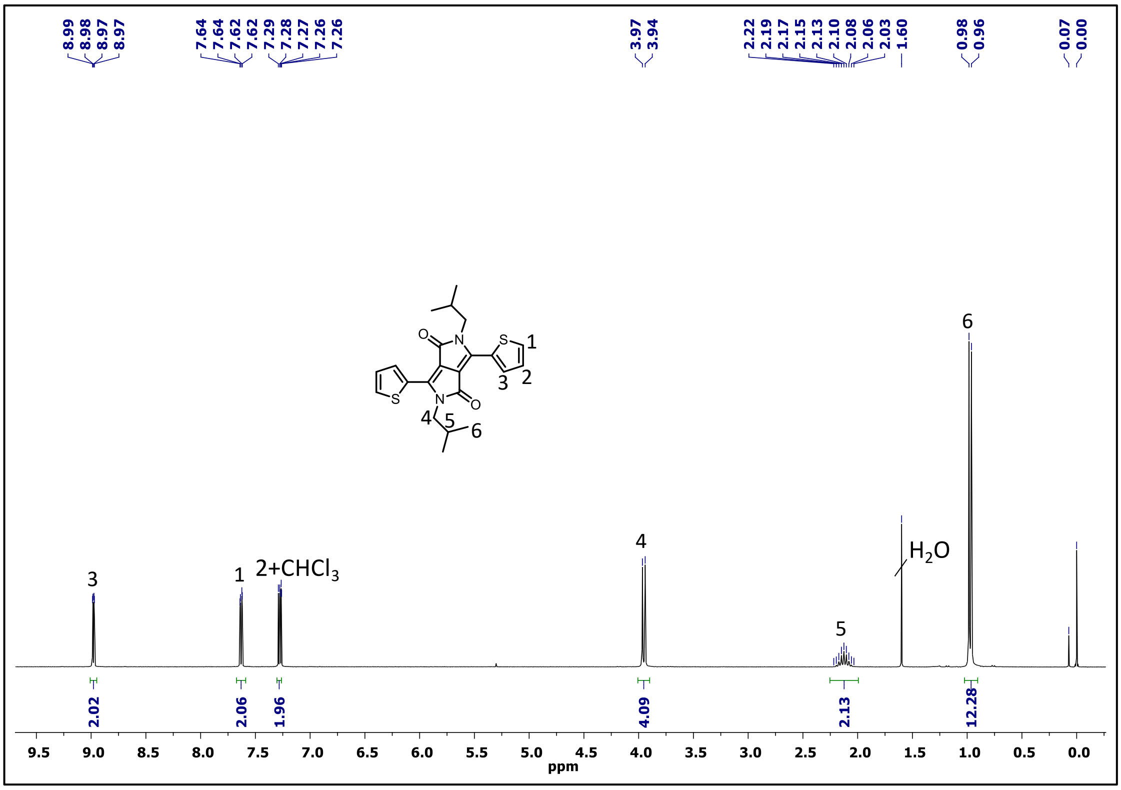

Figure S23. ^{1}H NMR of **DPP-Ref** ($CDCl_3$ at 25 °C).

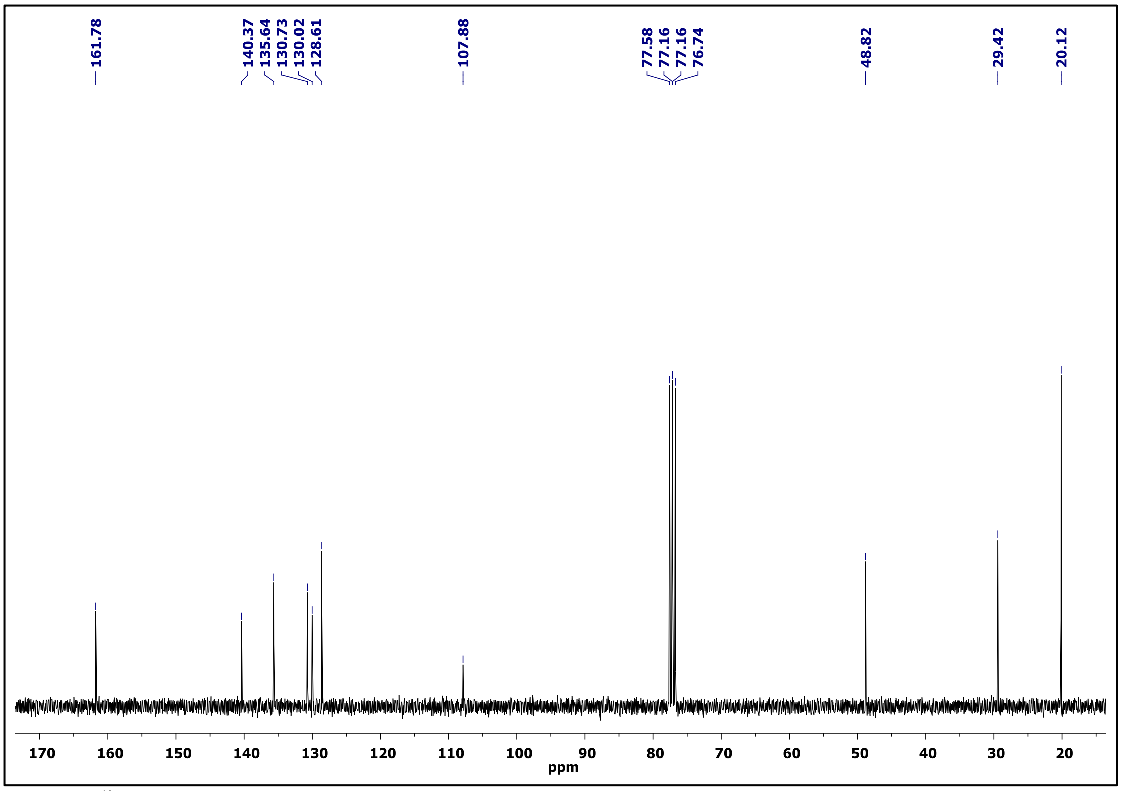

Figure S24. ^{13}C NMR of **DPP-Ref** ($CDCl_3$ at 25 °C).

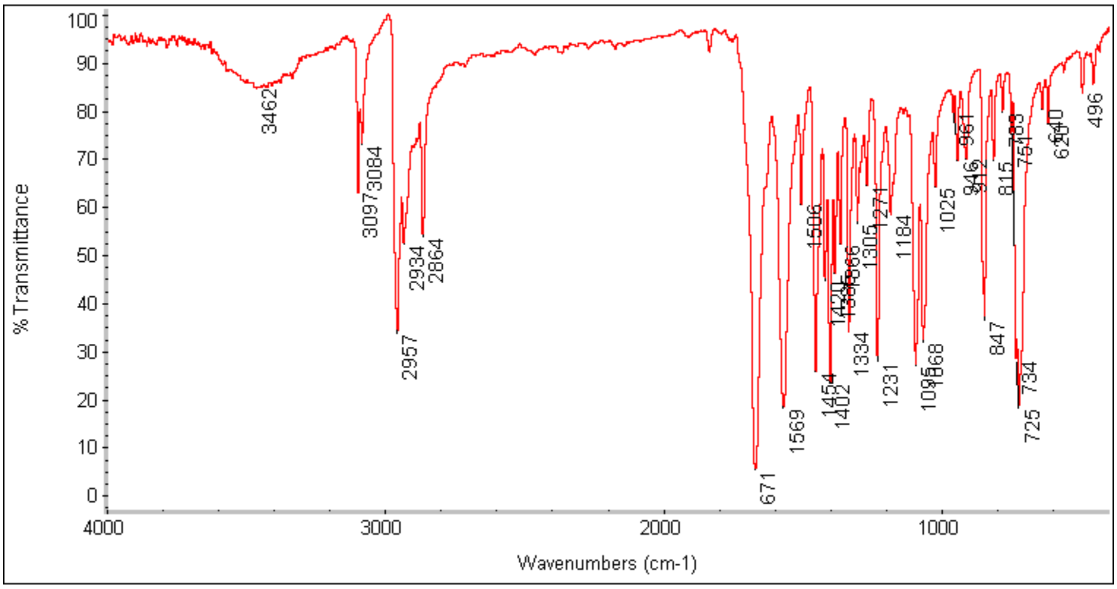

Figure S25. FTIR of **DPP-Ref**.

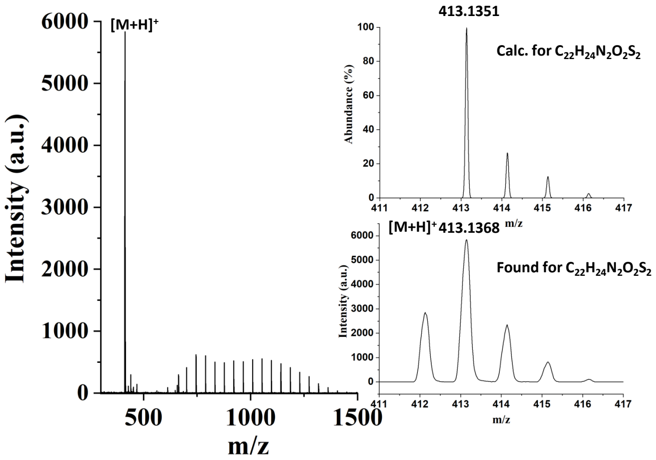

Figure S26. HR-MALDI-TOF of **DPP-Ref**.

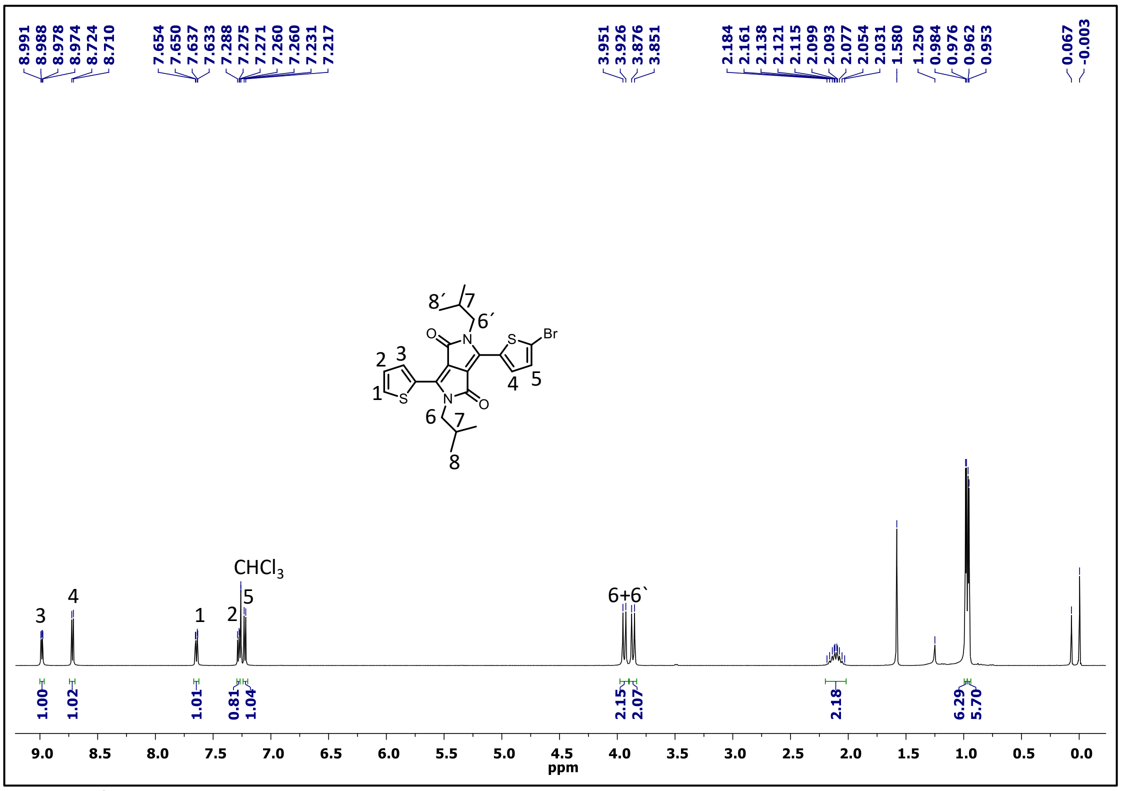

Figure S27. ^{1}H NMR of **DPP-Br** ($CDCl_3$ at 25 °C).

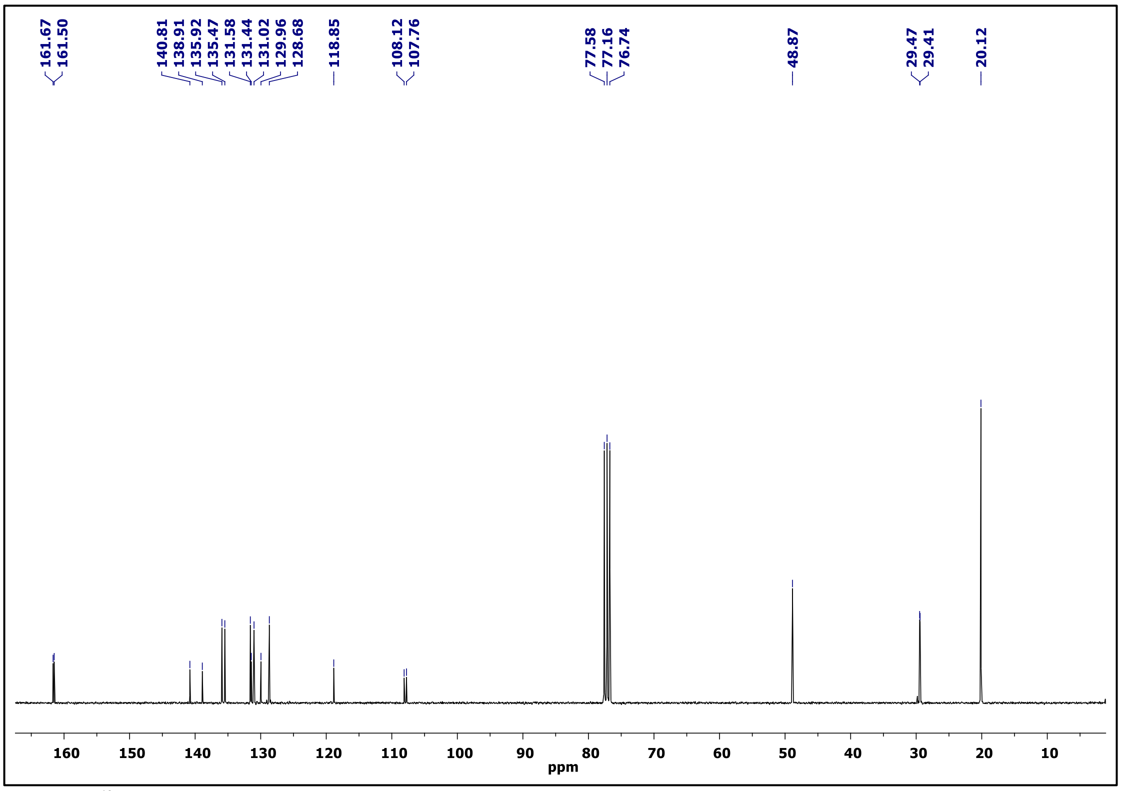

Figure S28. ^{13}C NMR of **DPP-Br** ($CDCl_3$ at 25 °C).

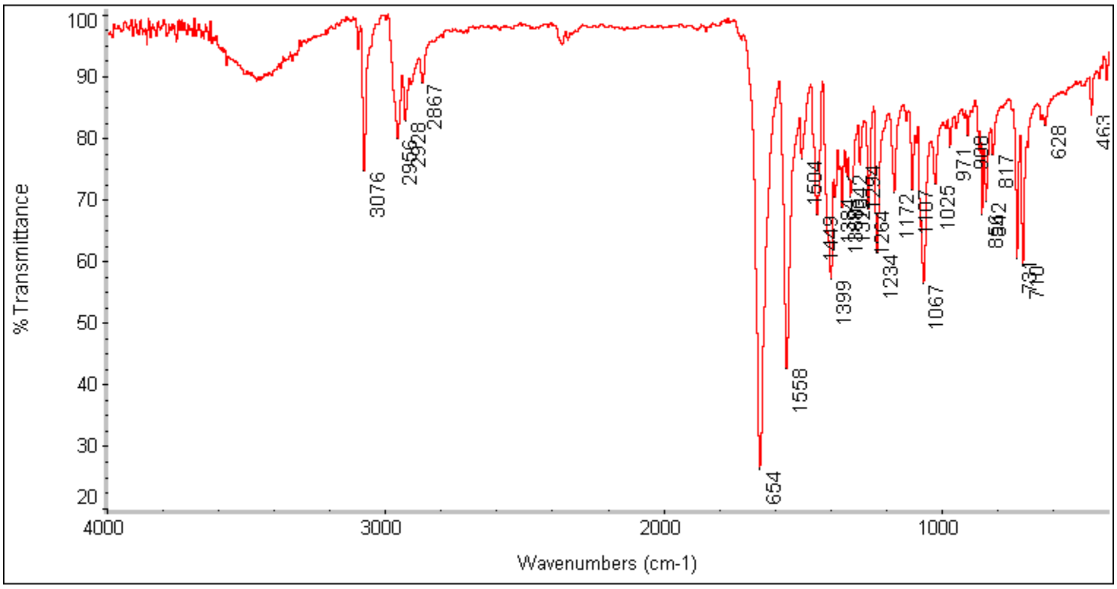

Figure S29. FTIR of **DPP-Br**.

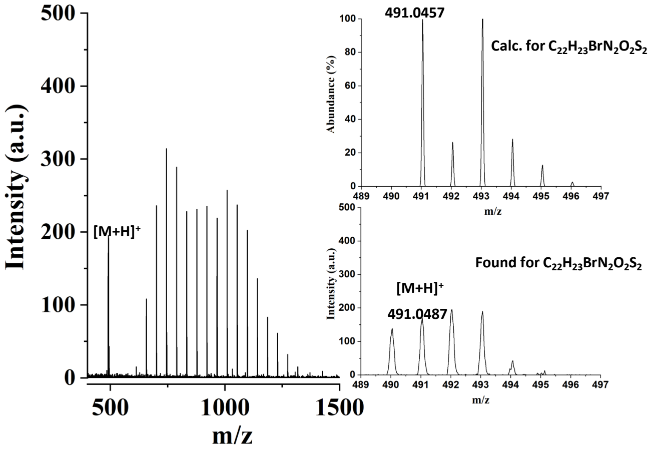

Figure S30. HR-MALDI-TOF of **DPP-Br**.

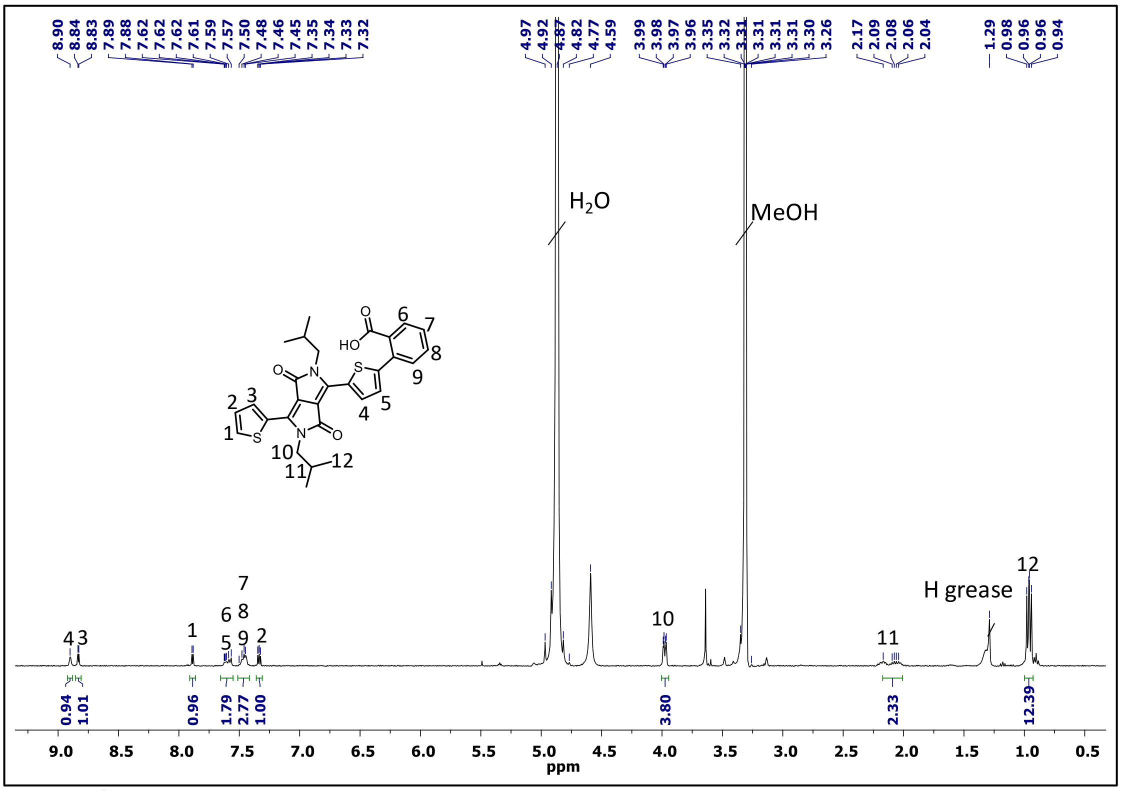

Figure S31. ^{1}H NMR of ***o*-DPP** (MeOH-d4 at 25 °C).

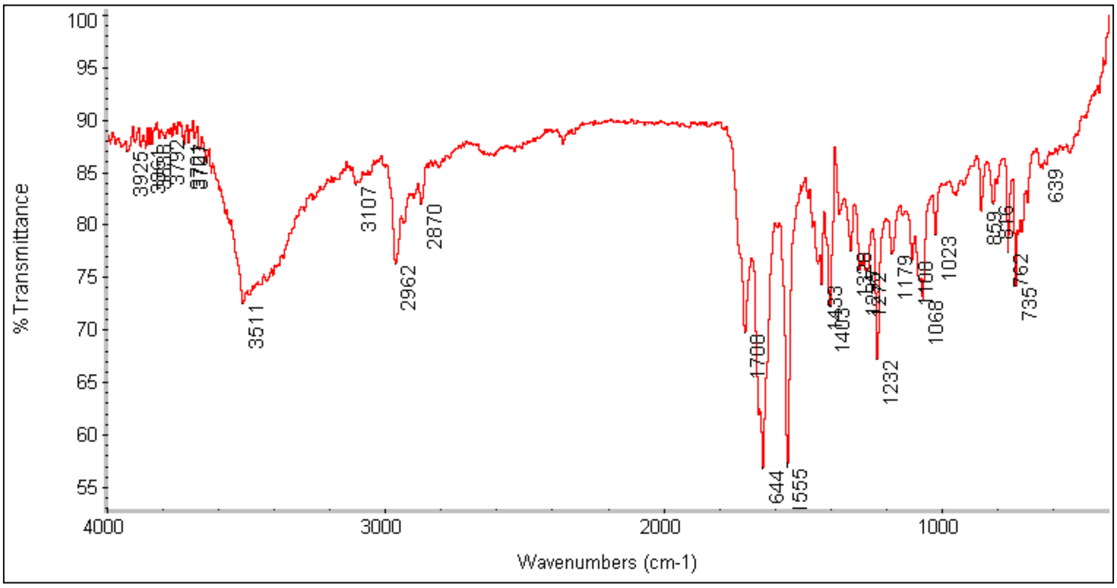

Figure S32. FTIR of ***o*-DPP**.

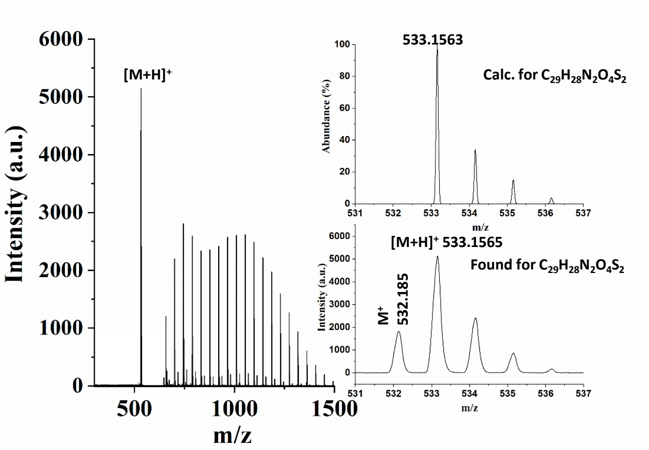

Figure S33. HR-MALDI-TOF of ***o*-DPP**.

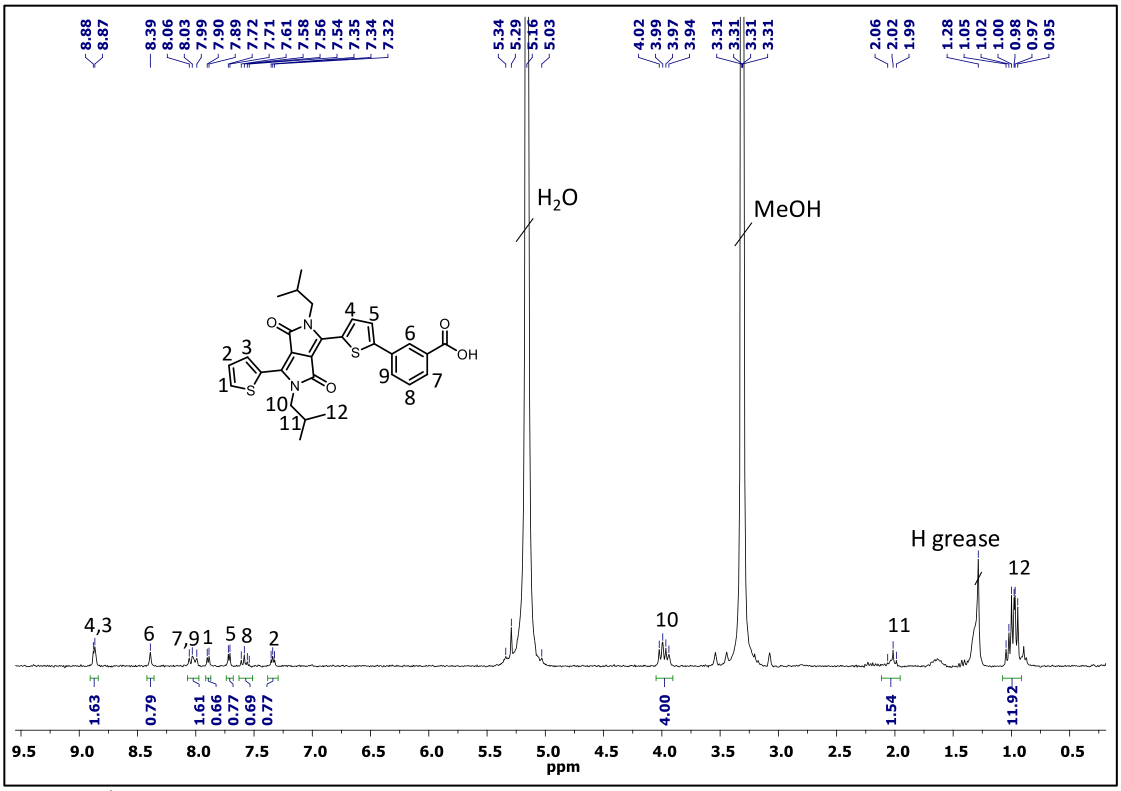

Figure S34. ^{1}H NMR of ***m*-DPP** (MeOH-d4 + 2 drops of TFA-d at 25 °C).

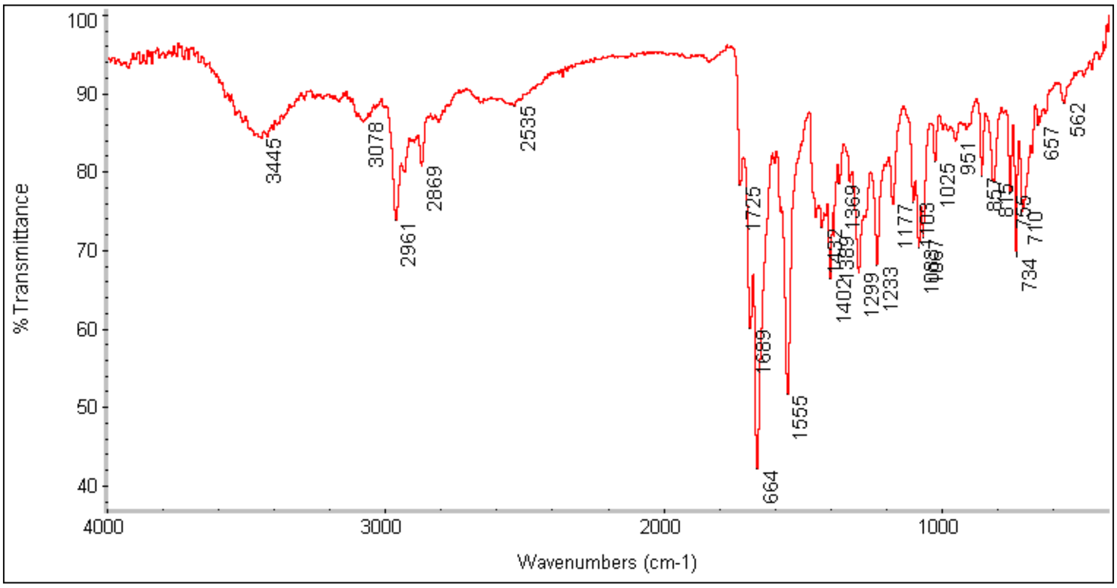

Figure S35. FTIR of ***m*-DPP**.

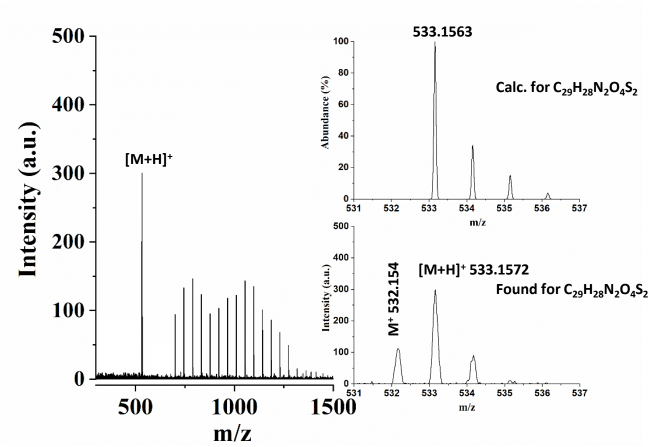

Figure S36. HR-MALDI-TOF of ***m*-DPP**.

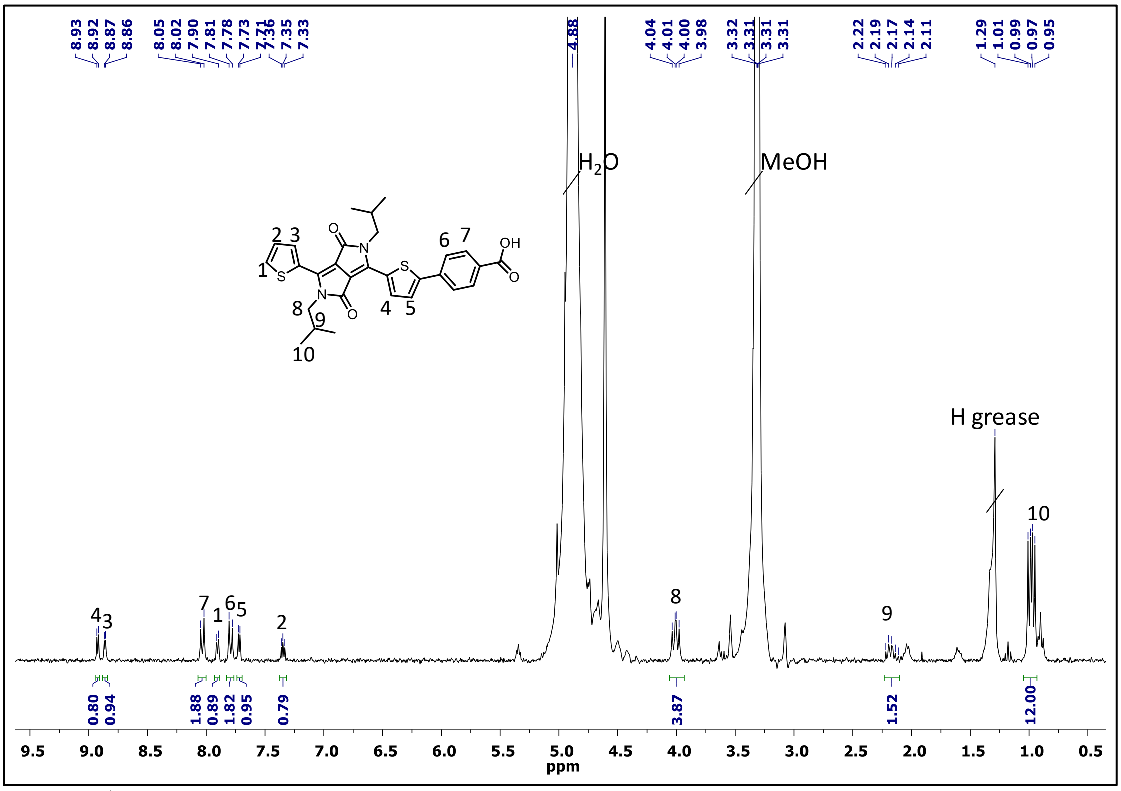

Figure S37. ^{1}H NMR of ***p*-DPP** (MeOH-d4 at 25 °C).

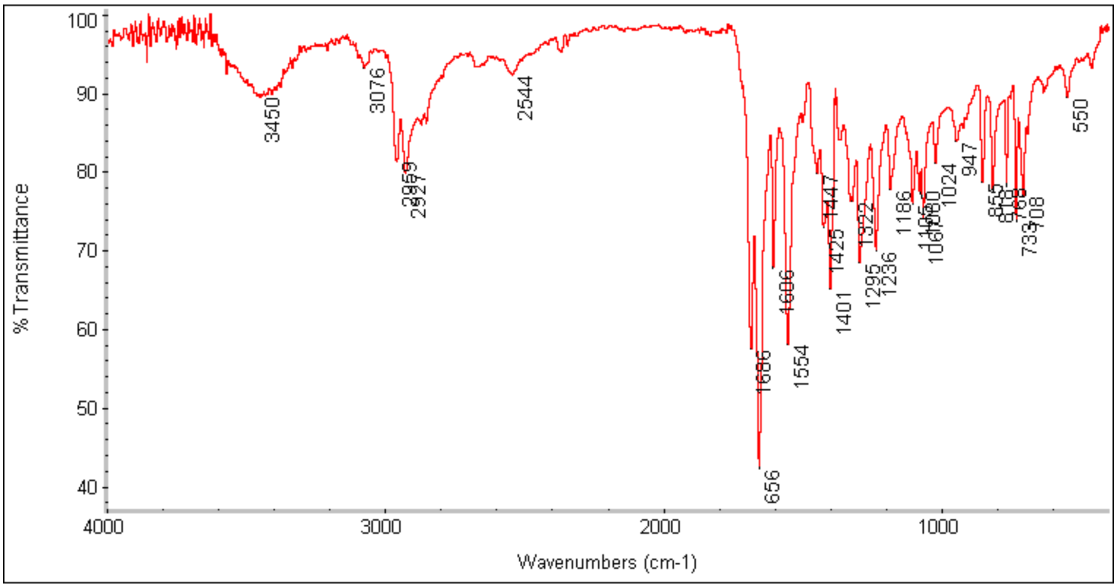

Figure S38. FTIR of ***p*-DPP**.

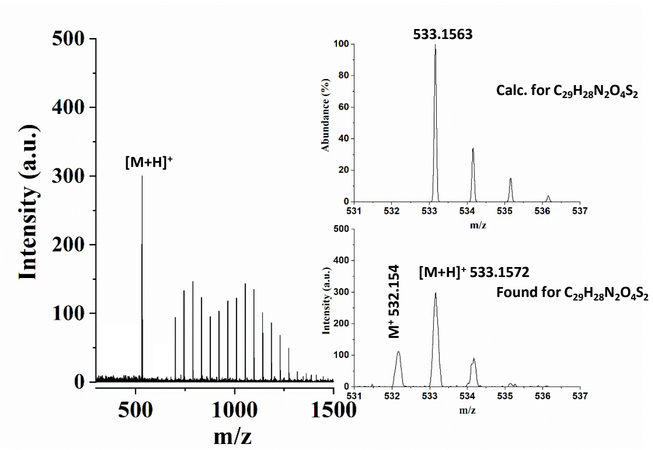

Figure S39. HR-MALDI-TOF of ***p*-DPP**.

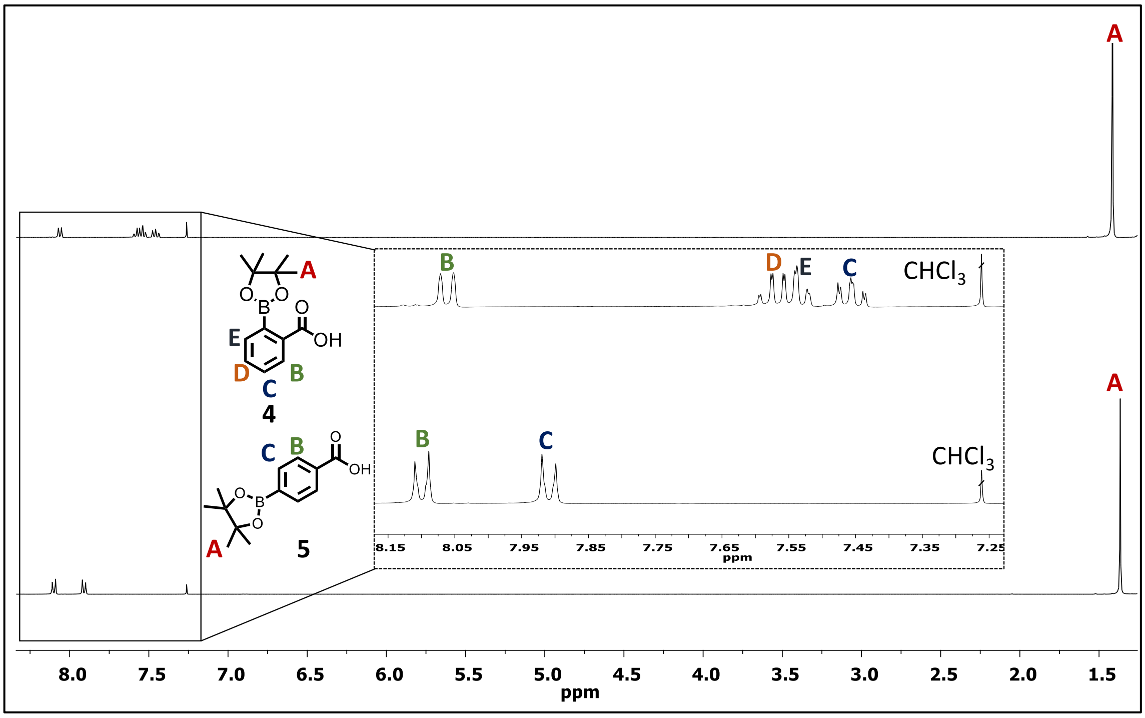

Figure S40. ^{1}H NMR of **1** and **3** in $CDCl_3$ at 25 **℃**.

1.5.2. Characterization of PMIDE derivatives

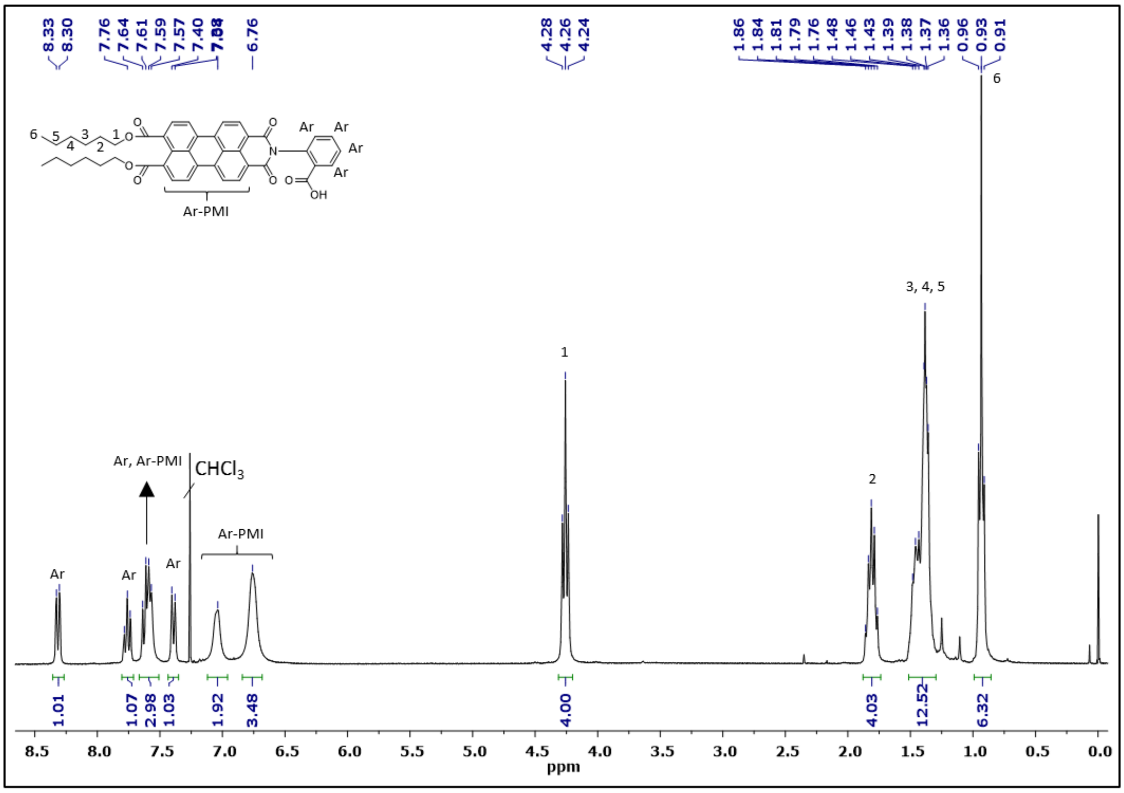

Figure S41. ^{1}H NMR of ***o*-PMIDE** ($CDCl_3$ at 25 °C).

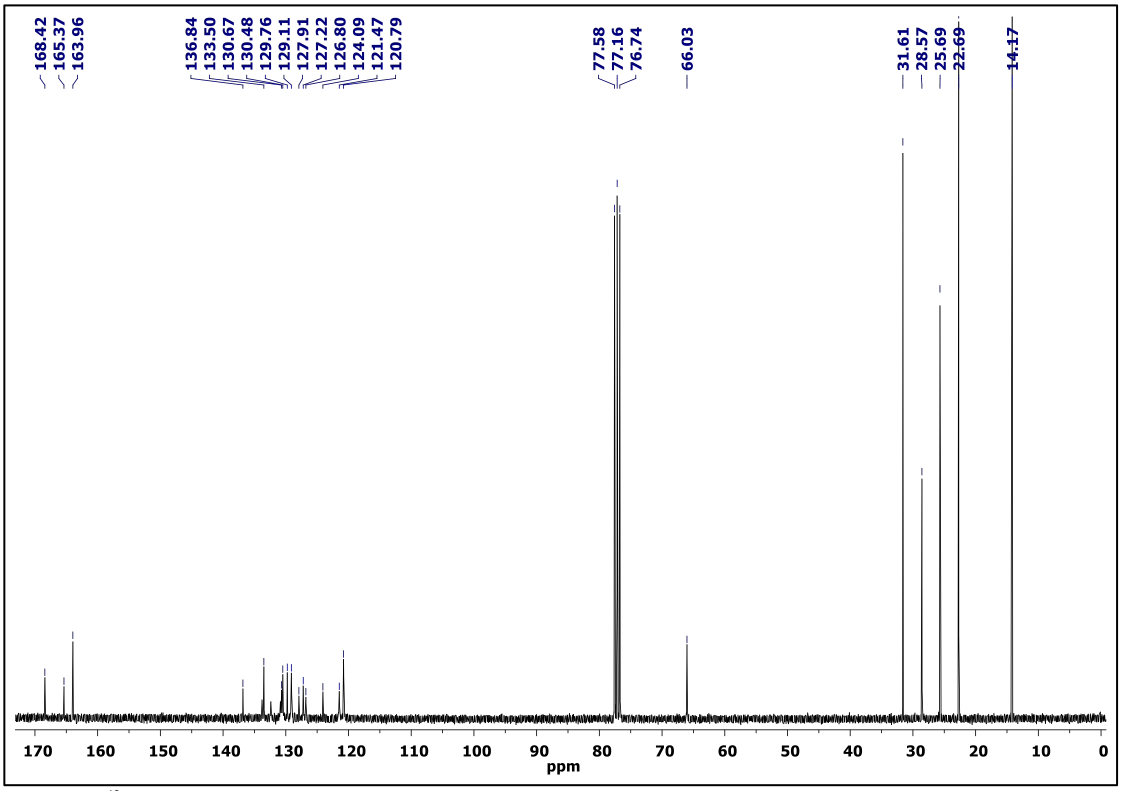

Figure S42. ^{13}C NMR of ***o*-PMIDE** ($CDCl_3$ at 25 °C).

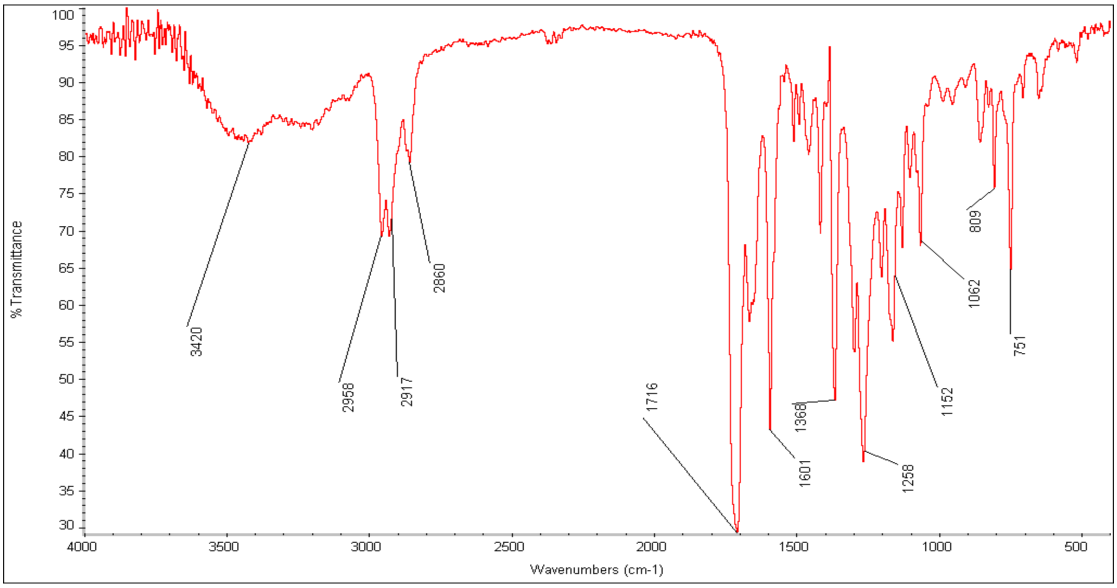

Figure S43. FTIR of ***o*-PMIDE**.

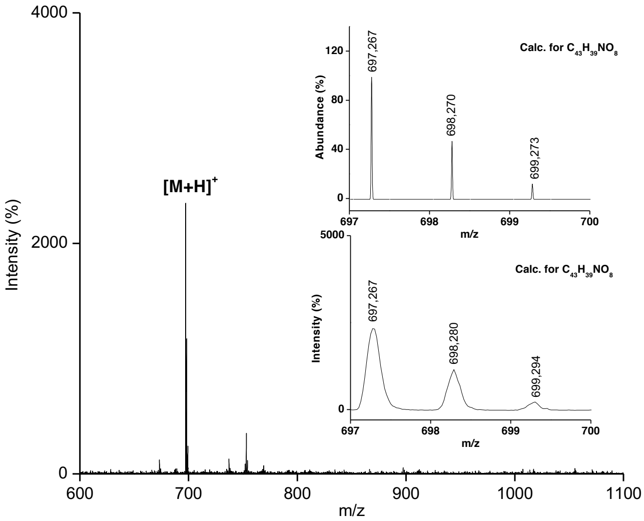

Figure S44. HR-MALDI-TOF of ***o*-PMIDE.**

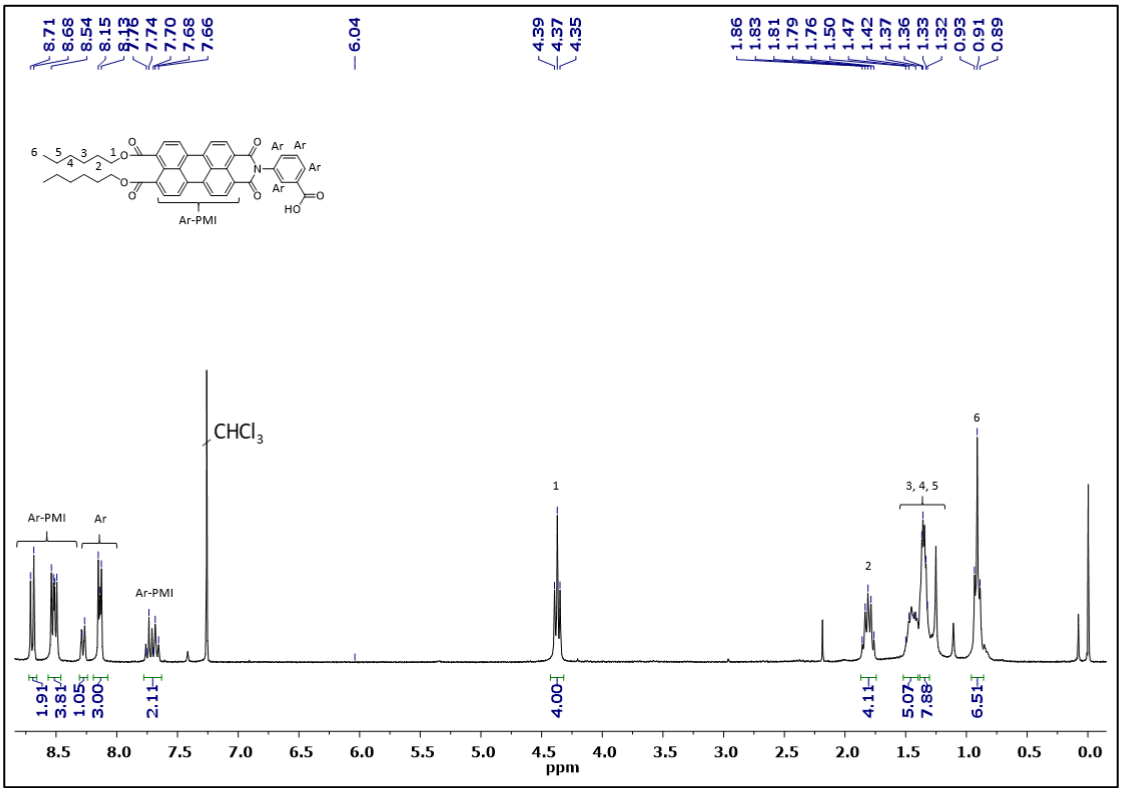

Figure S45. ^{1}H NMR of ***m*-PMIDE** ($CDCl_3$ + 1 drop TFA-d at 25 ℃).

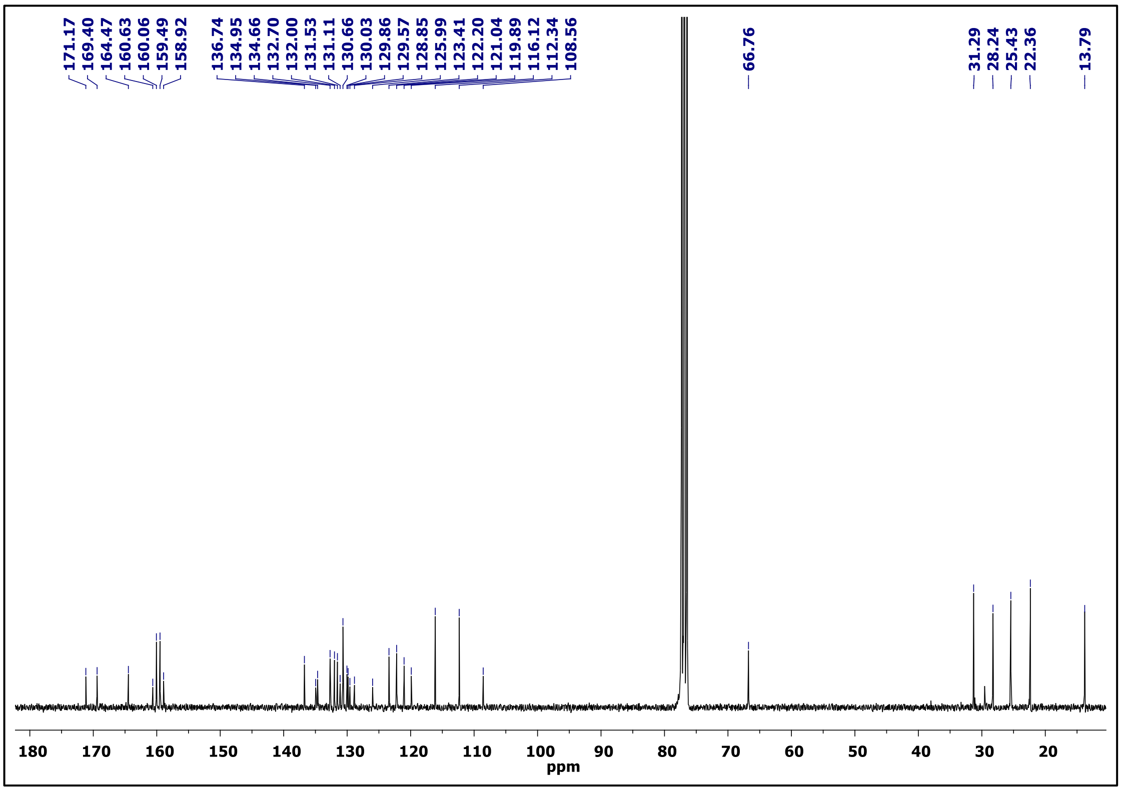

Figure S46. ^{13}C NMR of ***m*-PMIDE** ($CDCl_3$ + 1 drop TFA-d at 25 ℃).

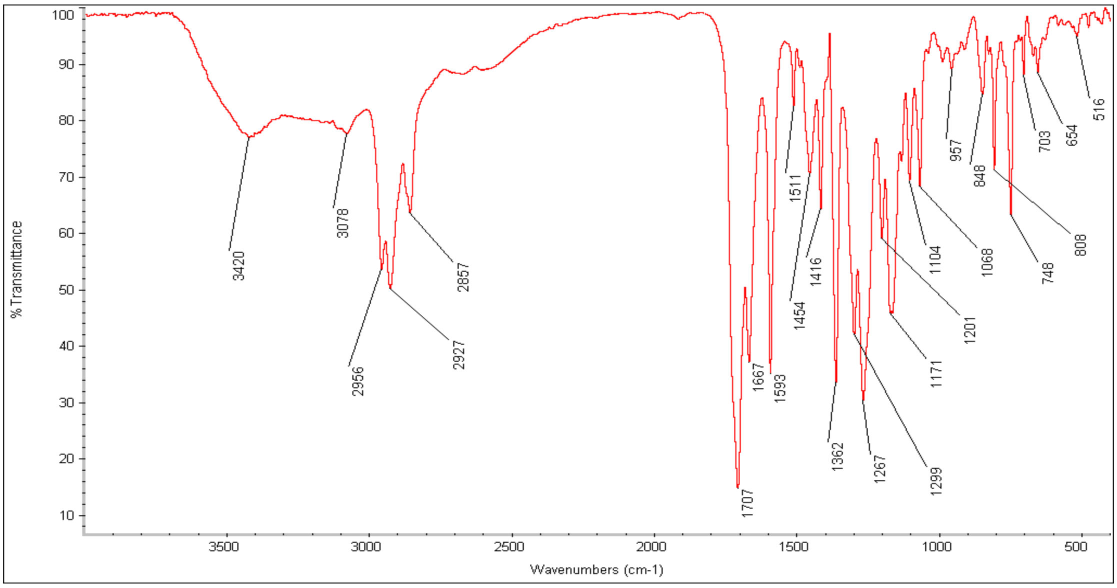

Figure S47. FTIR of ***m*-PMIDE**.

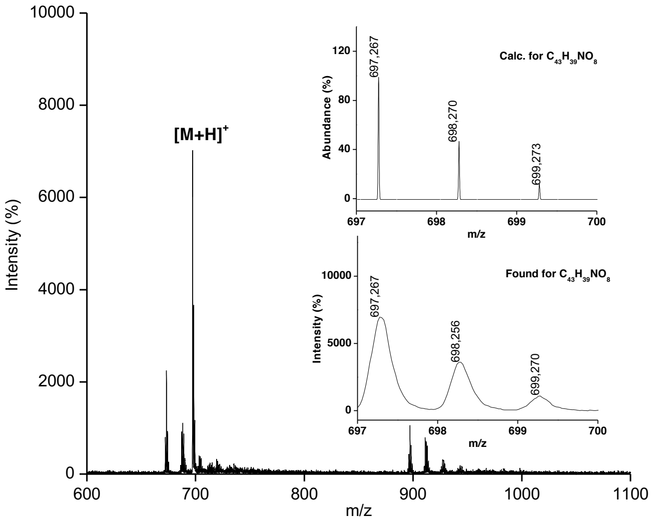

Figure S48. HR-MALDI-TOF of ***m-PMIDE***.

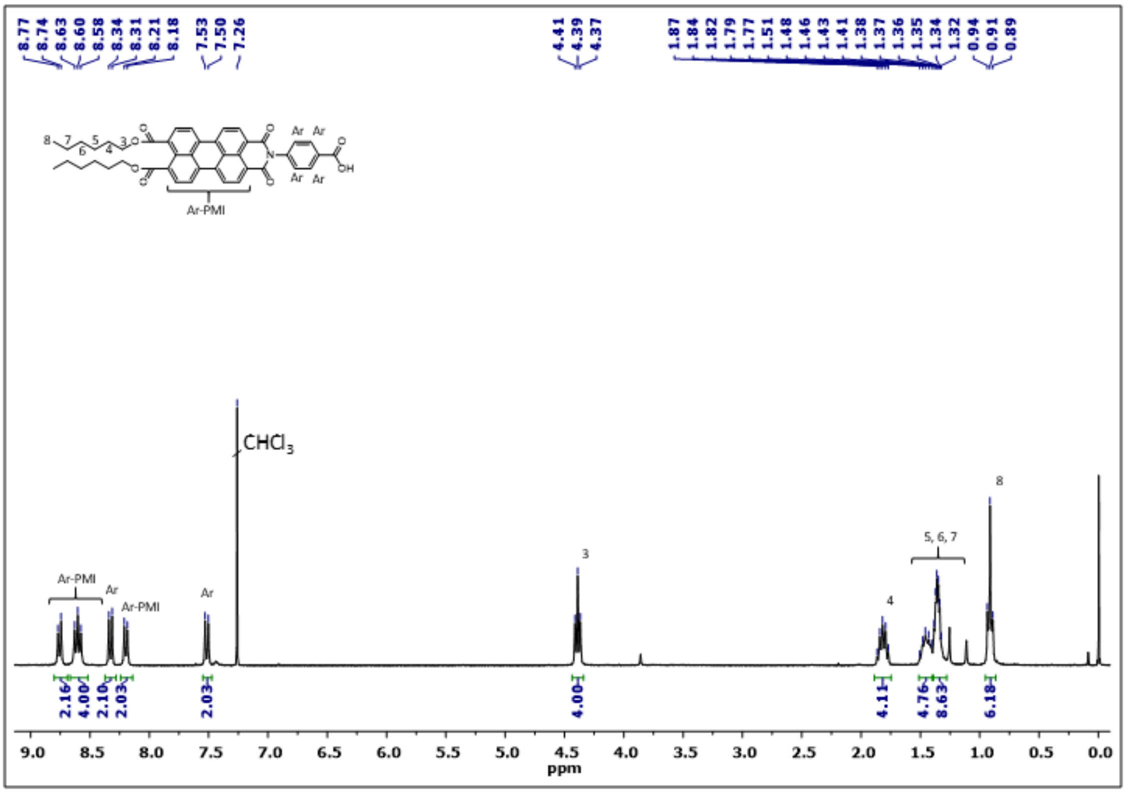

Figure S49. ^{1}H NMR of ***p*-PMIDE** ($CDCl_3$ + 1 drop TFA-d at 25 °C).

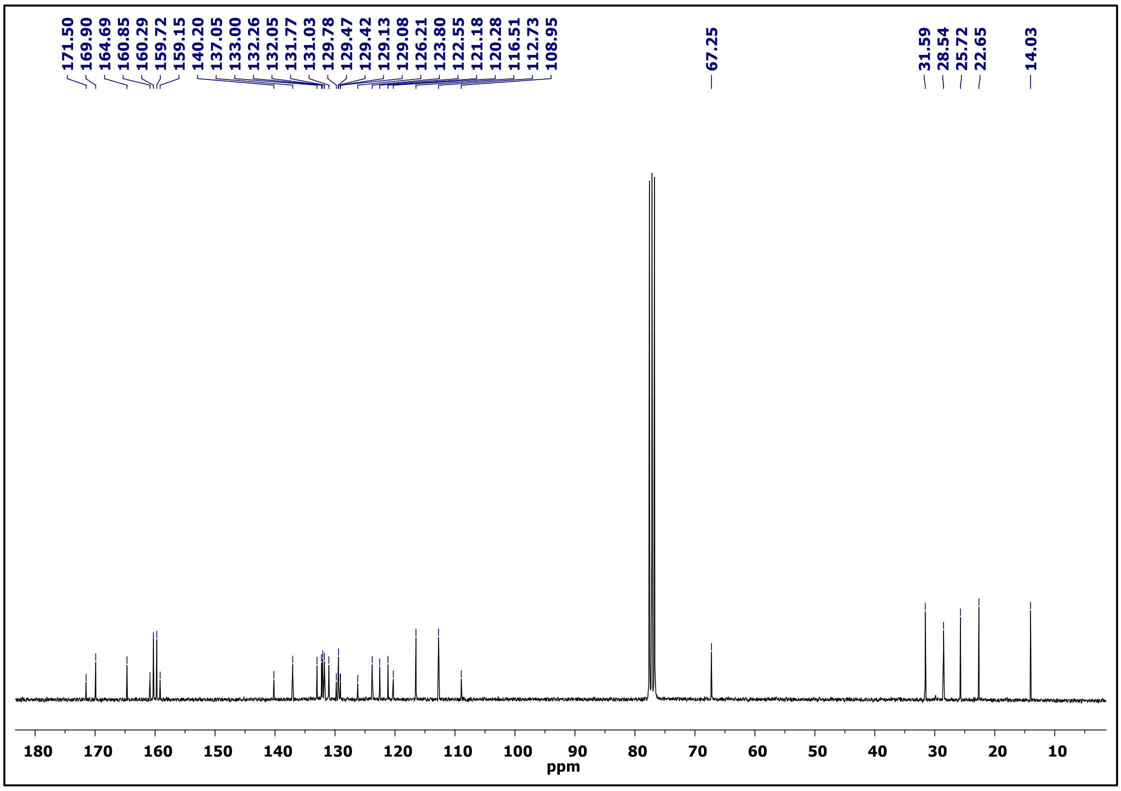

Figure S50. ^{13}C NMR of ***p*-PMIDE** ($CDCl_3$ + 1 drop TFA-d at 25 °C).

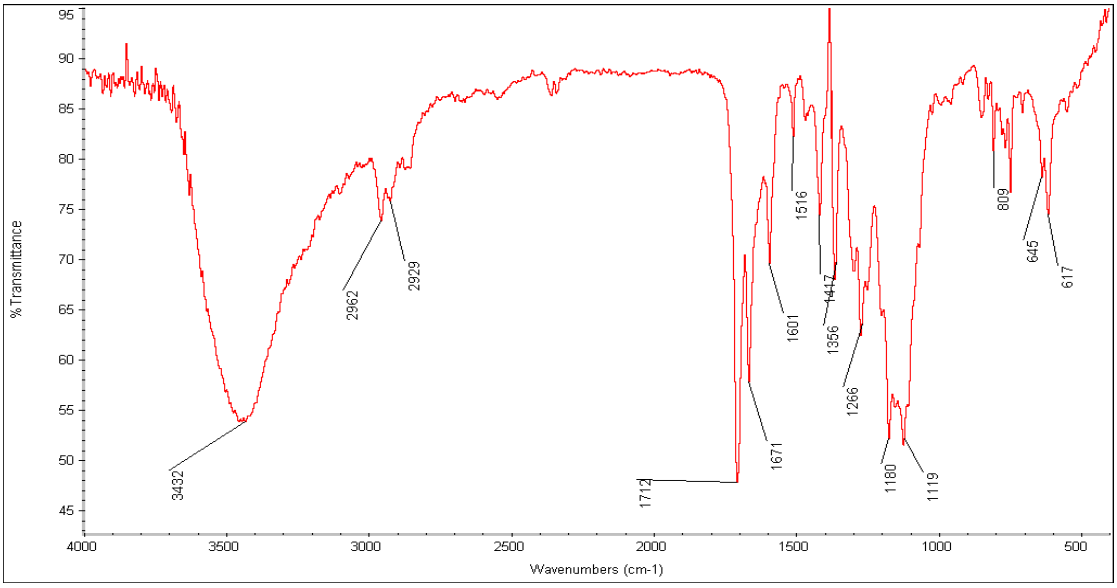

Figure S51. FTIR of ***p*-PMIDE**.

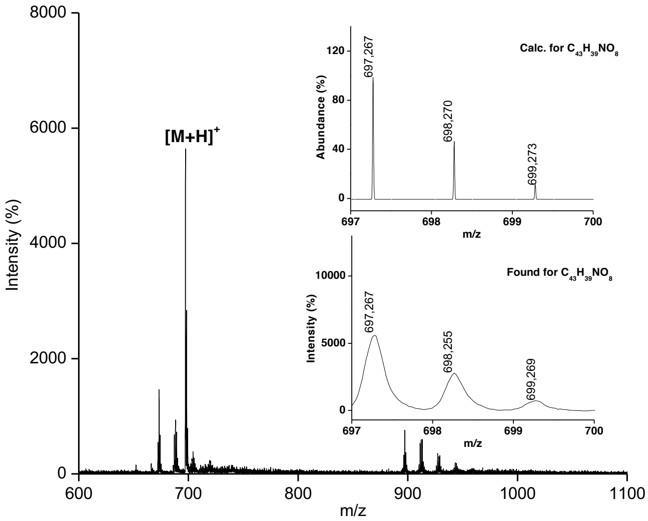

Figure S52. HR-MALDI-TOF of ***p*-PMIDE**.
